# Surface and morphology analyses, and voltammetry studies for electrochemical determination of cerium(iii) using a graphene nanobud-modified-carbon felt electrode in acidic buffer solution (pH 4.0 ± 0.05)†

**DOI:** 10.1039/d0ra07555h

**Published:** 2020-10-09

**Authors:** Pavithra V. Ravi, Daniel T. Thangadurai, Kasi Nehru, Yong Ill Lee, Devaraj Nataraj, Sabu Thomas, Nandakumar Kalarikkal, Jiya Jose

**Affiliations:** Department of Nanoscience and Technology, Sri Ramakrishana Engineering College, Affiliated to Anna University Coimbatore – 641 022 Tamilnadu India danielt@srec.ac.in; Department of Chemistry, Anna University – Bharathidasan Institute of Technology Tiruchirappalli – 620 024 Tamilnadu India; Department of Chemistry, Changwon National University Changwon 641-773 South Korea; Department of Physics, Bharathiar University Coimbatore – 641 046 Tamilnadu India; International and Inter-University Centre for Nanoscience and Nontechnology, Mahatma Gandhi University Kottayam – 686 560 Kerala India

## Abstract

Trace determination of radioactive waste, especially Ce^3+^, by electrochemical methods has rarely been attempted. Ce^3+^ is (i) a fluorescence quencher, (ii) an antiferromagnet, and (iii) a superconductor, and it has been incorporated into fast scintillators, LED phosphors, and fluorescent lamps. Although Ce^3+^ has been utilized in many industries due to its specific properties, it causes severe health problems to human beings because of its toxicity. Nanomaterials with fascinating electrical properties can play a vital role in the fabrication of a sensor device to detect the analyte of interest. In the present study, surfactant-free 1,8-diaminonaphthalene (DAN)-functionalized graphene quantum dots (DAN-GQDs) with nanobud (NB) morphology were utilized for the determination of Ce^3+^ through electrochemical studies. The working electrode, graphene nanobud (GNB)-modified-carbon felt (CF), was developed by a simple drop-coating method for the sensitive detection of Ce^3+^ in acetate buffer solution (ABS, pH 4.0 ± 0.05) at a scan rate of 50 mV s^−1^ using cyclic voltammetry (CV) and differential pulse voltammetry (DPV) techniques. CV and DPV studies validated the existence of distinctive peaks at approximately +0.20 and +0.93 V (*vs.* SCE), respectively, with a limit of detection of approximately 2.60 μM. Furthermore, electrochemical studies revealed that the GNB-modified-CF electrode was (i) stable even after fifteen cycles, (ii) reproducible, (iii) selective towards Ce^3+^, (iv) strongly pH-dependent, and (v) favored Ce^3+^ sensing only at pH 4.0 ± 0.05. Impedance spectroscopy results indicated that the GNB-modified-CF electrode was more conductive (1.38 × 10^−4^ S m^−1^) and exhibited more rapid electron transfer than bare CF, which agrees with the attained Randles equivalent circuit. Microscopy (AFM, FE-SEM, and HR-TEM), spectroscopy (XPS and Raman), XRD, and energy-dispersive X-ray (EDX) analyses of the GNB-modified-CF electrode confirmed the adsorption of Ce^3+^ onto the electrode surface and the size of the electrode material. Ce^3+^ nanobuds increased from 35–40 to 50–55 nm without changing their morphology. The obtained results provide an insight into the determination of Ce^3+^ to develop an electrochemical device with low sensitivity.

## Abstract 2

GNB-modified – CF electrode was utilized to determine Ce^3+^ with LoD *ca.* 2.60 μM.

## Graphical abstract

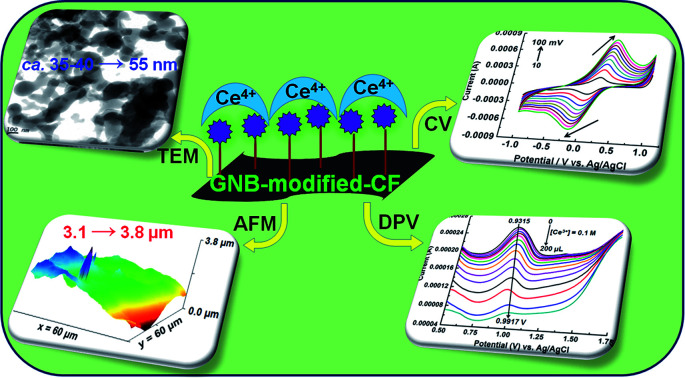

## Introduction

Cerium is one of the utmost copious elements of the lanthanum series and most widely distributed among the rare earth elements, with a presence in the Earth's crust at an average of 22 mg kg^−1^.^1,2^ Elemental cerium is an iron-gray, ductile, malleable metal. Cerium metal is very reactive and a strong oxidizing agent that is stabilized when connected with an oxygen ligand.^3^ Ce^3+^ is known as a fluorescence quencher, and due to its idiosyncratic properties, it has many potential applications in chemical and metallurgical engineering, agriculture, catalytic conversions, selective oxidation of hydrocarbons, nuclear energy, microelectronics, therapeutic application, and magnetism.^2,4,5^ Because it has been immensely used, there is an escalating demand to study the effects of cerium in medical, biological, and environmental applications.^6^

Accumulation toxicity is usually associated with cerium, and it affects the immune system, skin, bone organization, liver, heart, and central nervous system,^7^ and also leads to a host of diseases such as leukemia, skin lesions, acute myocardial infarction, and abnormal blood biochemical indices.^8,9^ Consequently, developing a fast, reliable, sensitive, and selective method for the detection of cerium is a real challenge for researchers around the globe.

Numerous techniques have been reported for the detection and quantitation of cerium, such as atomic absorption spectrophotometry, neutron activation analysis, inductively coupled plasma-atomic emission spectrometry, ion-selective electrodes, and potentiometric titration.^10–13^ These techniques have numerous disadvantages including high energy consumption, cost, diminutive repeatability, and complicated under-field conditions. Nevertheless, electrochemical techniques are considered as exceptional choices for the determination of trace modules due to their excellent selectivity, high sensitivity, easy handling, cost-efficacy, and quick response.^14^

The mechanism behind an electrochemical sensor facilitates the sensor to respond to the analyte in very little time, even with very low concentrations. Archetypal redox current peaks may assist with accurate qualitative detection to assure selectivity and anti-interference.^15^ Furthermore, very well-designed modified materials on the electrodes may enable ultra-low concentration detection of trace analytes.^16,17^ Because of its practicality and perfection, the electrochemical sensor is the most optimal method for Ce^3+^ detection. Therefore, the erudition of modified material to create a paradigm of an electrochemical sensor for the detection of Ce^3+^ with perfect selectivity and low detection limit is of great significance.^18^

Graphene quantum dots (GQD), which are implausibly small pieces of graphene, were discovered in the 20th century as a type of zero-dimensional material that can have lateral dimensions less than 100 nm in a single layer, double layers, and a few layers (3 to <10).^19–21^ As a result of converting two-dimensional graphene sheets into zero-dimensional GQD, the GQDs exhibit new phenomena due to quantum confinement and edge effects.^22^ Because of the superior properties of GQDs, such as low cytotoxicity and biocompatibility, chemical inertness, and resistance to photobleaching, they are propitious candidates for optoelectronic devices, bioimaging, and sensors.^21,23^ Additionally, GQDs have outstanding characteristics of fine surface grafting using the π–π conjugated network or surface groups, large surface area and diameter, and other unique physical properties.^24^ Furthermore, the COO^−^ and OH^−^ present at their edges enable the GQDs to be remarkably water soluble, and thus appropriate for consecutive functionalization with various organic, inorganic, polymeric, or biological components.^23,25,26^ However, there have been few reports available describing the functionalization of GQDs with organic molecules or polymers for sensing applications, and none of them have demonstrated Ce^3+^ detection.^24,27–31^

Based on the above exploration and continuation of our study on multifunctional sensors,^23,25,32–34^ herein, we report the detection of Ce^3+^ using a graphene nanobud (GNB)-modified-carbon felt (CF) electrode as a working electrode in acetate buffer solution (ABS) at a pH of 4.0 ± 0.05 using cyclic voltammetry (CV) and differential pulse voltammetry (DPV) techniques. We also evaluated the conducting nature of GNB-modified-CF electrodes by impedance spectroscopy and electrochemical performance with (i) a fixed concentration of Ce^3+^ (0.1 M) and a (ii) fixed scan rate (50 mV s^−1^), and also evaluated the (iii) effect of pH, (iv) effect of time, and (v) interference of other metal ions by CV studies.

## Results and discussion

Qualitative and quantitative detection of radioactive wastes, which contain certain biological and chemical residues, is very important to define an appropriate management process.^35^ Because of the disadvantages of currently existing competing analytical techniques, developing a simple sensing method to detect and quantify these wastes is important to ensure safety. However, the electrochemical method is economical, more suitable and convenient, highly selective and sensitive, and reproducible.^36,37^ The well-designed and structurally modified materials on the electrodes can promote the enhancement of trace analytes to attain an ultra-low concentration detection.^18^ The electrode material, 1,8-diaminonaphthalene (DAN)-GQD, in nanobud morphology was synthesized without using any surfactant in an aqueous medium and was systematically characterized by standard physiochemical techniques,^25^ which is shown in the ESI (Fig. SI1).†

**Fig. 1:**
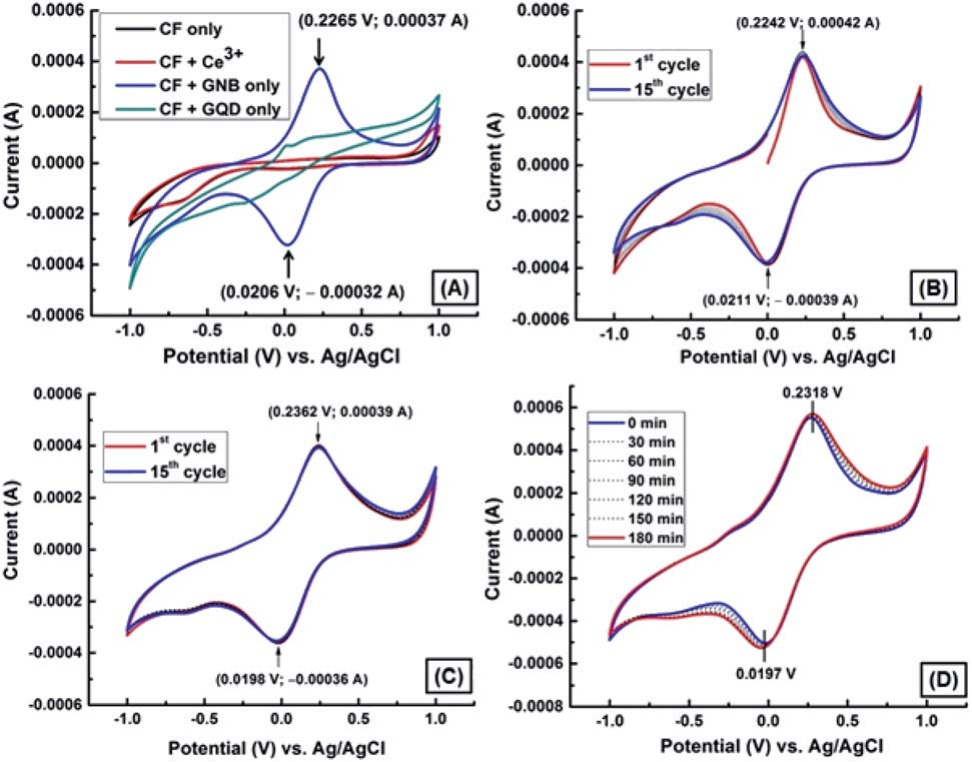
(A) CV obtained for CF (black line), CF + Ce^3+^ (red line), the GNB-modified-CF electrode without Ce^3+^ (blue line), and CF + GQD (green line). (B) CV obtained for the GNB-modified-CF electrode without Ce^3+^, first cycle (red line) and fifteenth cycle (blue line). (C) CV obtained for GNB-modified-CF with Ce^3+^ (0.1 M; 10 μL), first cycle (red line) and fifteenth cycle (blue line). (D) CV obtained for the GNB-modified-CF electrode with Ce^3+^ (0.1 M; 10 μL) in every 30 minute time interval in ABS (pH 4.0 ± 0.05) at a scan rate of 50 mV s^−1^.

Because of the negative health effects of Ce^3+^ on humans, we decided to carry out electrochemical experiments (CV and DPV) to detect Ce^3+^ using a GNB-modified-CF electrode in ABS (pH 4.0 ± 0.05) at a scan rate of 50 mV s^−1^. The fabrication of the working electrode, a GNB-modified-CF electrode, is illustrated in Fig. SI2.† The electrochemical behavior of the GNB-modified-CF electrode without and with Ce^3+^ was examined (Fig. 1A–C). No characteristic peak was observed for the bare CF electrode without and with Ce^3+^ in the potential region from −1.0 to 1.0 V (black and red line, respectively; Fig. 1A). However, the GNB-modified-CF electrode exhibited an oxidation peak at approximately 0.236 V and a corresponding reduction peak at approximately 0.020 V upon addition of Ce^3+^ (0.1 M; 10 μL) in the potential region from −1.0 to 1.0 V (Fig. 1C). The electrochemical response of the GNB-modified-CF electrode without and with Ce^3+^ was stable even after fifteen cycles (Fig. 1B and C, respectively), which indicates the high stability of the working electrode. This high stability obtained with Ce^3+^ might infer that an easy electron transfer from the highly conducting GNB-modified-CF electrode to Ce^3+^ stopped the deterioration of the electrode surface caused by the formation of Ce^3+^ complex.^38^

These initial CV studies indicate that the GNB-modified-CF electrode can substantially promote electron transfer as well as increase the over potential for the redox of Ce^3+^ by approximately 215 mV when compared to bare CF, which does not show any noticeable redox response in the presence of Ce^3+^ (red line; Fig. 1A). Thus, this modified working electrode could be expedient for the quantitative detection of Ce^3+^ at acidic pH.

To validate the dependability of the GNB-modified-CF electrode, we examined its electrochemical behavior with Ce^3+^ (0.1 M; 10 μL) in 30 minute intervals in ABS (pH 4.0 ± 0.05) at a scan rate of 50 mV s^−1^. Fig. 1D illustrates that even after more than seven repeated analyses, the redox peak potentials remain comparable with a diminutive peak shift of less than 2.0%, due to the successive electron transfer reaction between Ce^3+^ and the GNB-modified-CF electrode. The obtained CV results confirm that the reported modified working electrode has excellent reproducibility, and the electrode surface was not worsened or debilitated during its activity even after 3 h.^38^ After examining all of the above electrochemical results, we concluded that the procedure for fabricating the current working electrode system requires less time, and is cost-effective, extremely stable, selective, sensitive, and reproducible towards Ce^3+^.

**Fig. 2:**
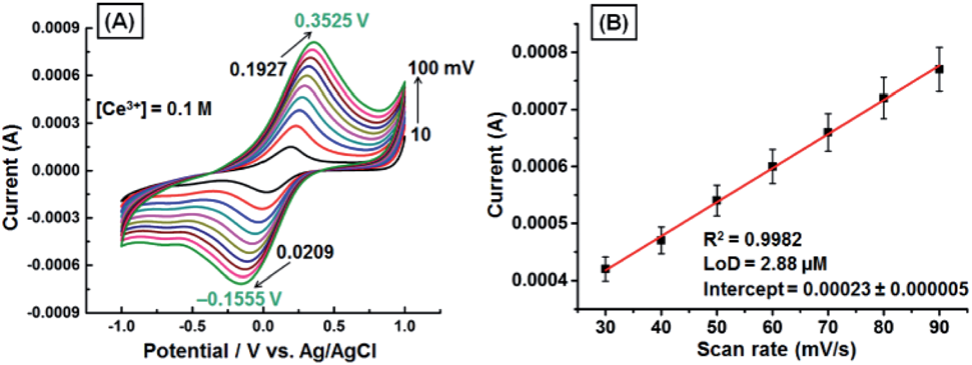
(A) CV obtained for the GNB-modified-CF electrode with Ce^3+^ (0.1 M; 10 μL) in ABS (pH 4.0 ± 0.05) at different scan rates (10–100 mV s^−1^). (B) Influence of scan rate on reduction peak current. Error bars: standard deviation of three independent measurements made from a newly prepared GNB-modified-CF electrode. Corresponding linear fit parameters can be found in Fig. SI3.†

The effect of scan rate on redox peak current of the GNB-modified-CF electrode upon addition of Ce^3+^ (0.1 M; 10 μL) in ABS (pH 4.0 ± 0.05) was examined under the scan rate of 10 to 100 mV s^−1^. The cyclic voltammogram as a function of voltage (*V*) *vs.* current (*I*) shows the shift of peak potential towards increasingly positive (approximately 160 mV) and increasingly negative (approximately 175 mV) in the reverse scan upon increasing the scan rate (Fig. 2A). The electrochemical behavior was found to be quasi-reversible in ABS at pH 4.0 ± 0.05, and both the oxidation and reduction peak currents were increased with increasing scan rate. The redox peak currents exhibited excellent linearity with scan rate (*R*^2^ = 0.9982), and the calculated limit of detection (LoD) was found to be approximately 2.88 μM (Fig. 2B). These higher potential shifts and increase in current intensity as a function of scan rates may be ascribed to diffusion-controlled processes and satisfactory adhesion.^39^

**Fig. 3:**
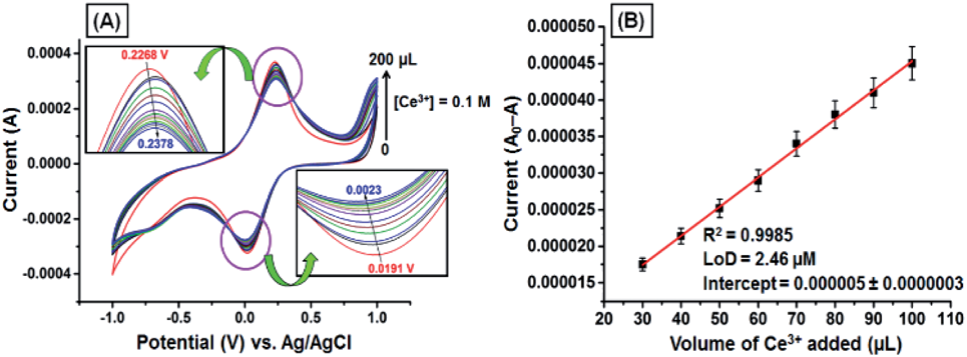
(A) CV obtained for the GNB-modified-CF electrode with Ce^3+^ (0.1 M; 0 → 200 μL) in ABS (pH 4.0 ± 0.05) at a scan rate of 50 mV s^−1^. (B) Influence of the volume of Ce^3+^ on the reduction of peak current. Error bars: standard deviation of three independent measurements made from a newly prepared GNB-modified-CF electrode. Corresponding linear fit parameters can be found in Fig. SI4.†

The CV responses of the GNB-modified-CF electrode upon various additions of Ce^3+^ (0.1 M; 0 → 200 μL) in ABS (pH 4.0 ± 0.05) at a scan rate of 50 mV s^−1^ are shown in Fig. 3. Upon addition of Ce^3+^ (0 → 200 μL), the oxidation potential shifted towards increasingly positive from 0.227 to 0.238 V (by approximately 11 mV), and reduction potential shifted towards decreasingly positive from 0.019 to 0.002 V (by approximately 17 mV) with decreasing peak current (Fig. 3A). These redox couple shifts are possibly due to stronger π–π interactions between the antiparallel aromatic moieties (naphthalene) present in the GNB-modified-CF electrode when compared with bare CF.^40^

Excellent linearity was observed while plotting peak current against the increasing volume of Ce^3+^, with a correlation coefficient of 0.9985 and an LoD of approximately 2.46 μM (Fig. 3B). This result indicates that the GNB-modified-CF electrode can appreciably promote electron transfer by increasing the over potential (approximately 28 mV) for the reduction of Ce^3+^ compared to bare CF, which is in accordance with our previous results. The above-mentioned CV results confirm that the GNB-modified-CF electrode could be convenient for the quantitative detection of Ce^3+^ at acidic pH.

**Fig. 4:**
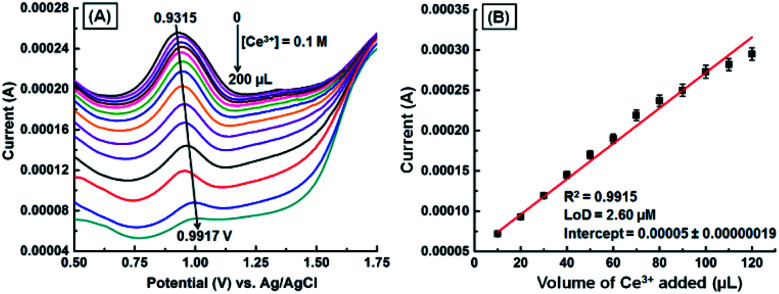
(A) DPV obtained for the GNB-modified-CF electrode with Ce^3+^ (0.1 M; 0 → 200 μL) in ABS (pH 4.0 ± 0.05) at a scan rate of 10 mV s^−1^. (B) Influence of volume of Ce^3+^ on peak current. Error bars: standard deviation of three independent measurements made from a newly-prepared GNB-modified-CF electrode. Corresponding linear fit parameters can be found in Fig. SI6.† The DPV obtained for CF with Ce^3+^ (0.1 M) can be found in Fig. SI5.†

A DPV study was carried out with the GNB-modified-CF electrode in ABS (pH 4.0 ± 0.05) at a scan rate of 10 mV s^−1^ (Fig. 4), with the intention of detecting Ce^3+^ upon its addition (0.1 M; 10 μL each addition). The bare CF showed an oxidation peak at +0.927 V and peak current at 0.00024 A in the potential range from 0.50 to 1.50 V (Fig. SI5†). The GNB-modified-CF electrode exhibited a distinct oxidation peak at +0.932 V, which indicated a very small shift to a high potential (+0.992 V), and the peak current decreased (0.00026 → 0.000007 A) upon addition of Ce^3+^ (0 → 200 μL) (Fig. 4A). The reason for this small shift of the oxidation peak might be due to the disruption of mass transport processes. In other words, to compensate for the restoration of mass transport and the diffusion problem, the electrochemical system applies more potential, which, in turn, results in a shift in peak potential.^40^ The calibration curve for the quantification of Ce^3+^ was constructed by plotting the dependence of peak current *vs.* increasing volume of Ce^3+^ (Fig. 4B). A comparative decrease in current (*I*) values with increasing volume of Ce^3+^ was observed (*R*^2^ = 0.9915), and from the DPV data, the calculated LoD was found to be approximately 2.60 μM.

**Fig. 5:**
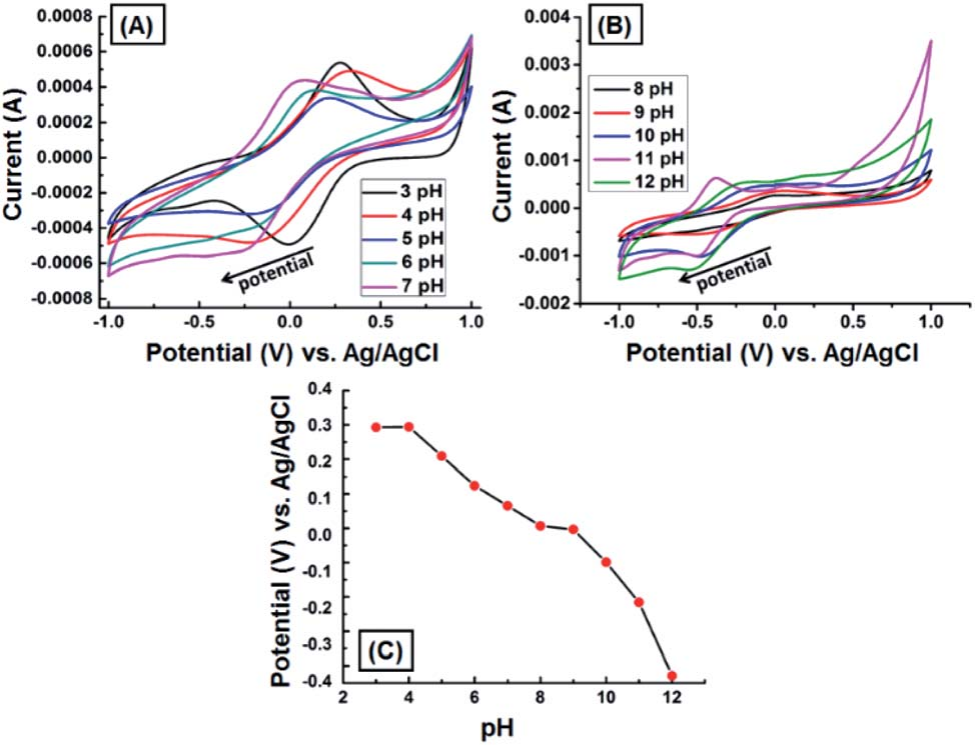
CV obtained for the GNB-modified-CF electrode with Ce^3+^ (0.1 M) in ABS (pH 4.0 ± 0.05) at pH ranging from (A) 3 to 7 and (B) 8 to 12 under the scan rate of 50 mV s^−1^. (C) Influence of pH on peak potential (plot of pH *vs.* potential).

We assessed the electrochemical behavior of the GNB-modified-CF electrode at different pH by conducting the CV measurement with Ce^3+^ (0.1 M) in 100 mL of electrolyte (neutral buffer solution prepared by using commercially available capsules) at pH ranging from 3 to 12 under the scan rate of 50 mV s^−1^. Fig. 5 illustrates that both peak potential and peak current were strongly pH-dependent. For instance, the peak potential sharply decreased (0.280 → −0.038 V) with increasing pH value (3 → 12), which is attributable to the decreasing concentration of H^+^ ions in the electrolyte (Fig. 5A and B). The peak current arbitrarily varied due to the interaction of inorganic cation, Ce^3+^, with the surface functional groups present on the GNB-modified-CF electrode.^41^ Importantly, the variation in the current at basic pH is perhaps due to the formation of metal hydroxide, which causes interference or change in the oxidation state of metal ion from Ce^3+^ to Ce^4+^.^42,43^ However, previous reports corroborate that metal cation sensing is effective only at acidic pH.^44,45^ Therefore, we carried out all the electrochemical studies using 0.2 M ABS at pH 4.0 ± 0.05. From the above pH study results, we concluded that the pH of the electrolyte regulates the stability of the newly formed DAN-GQD·Ce^3+^ complex and, as a consequence, resulted in a variation in the peak current and peak potential.^46^

**Fig. 6:**
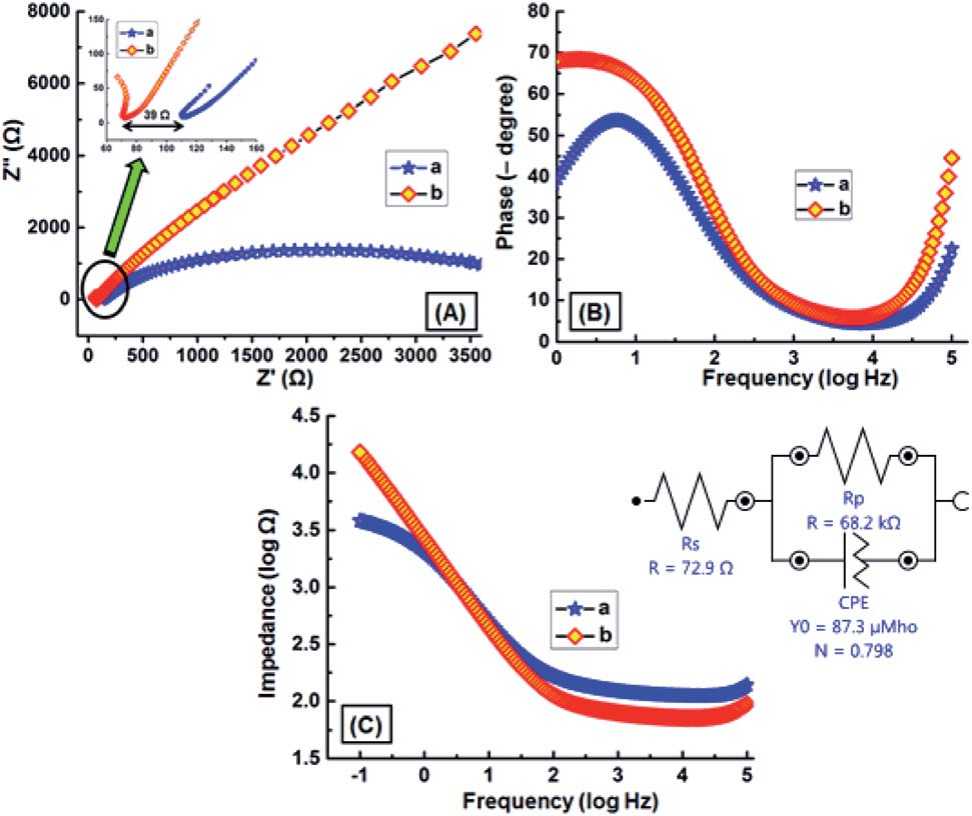
Impedance spectral data of (a) bare CF and (b) the GNB-modified-CF electrode in K_3_[Fe(CN)_6_] (2.0 mM) containing ABS (pH 4.0 ± 0.05) at scanning frequencies from 0.01 to 100 000 Hz. (A) Nyquist, (B) Bode-phase angle, and (C) Bode amplitude plots; (inset) equivalent electrical circuit used for fitting the impedance spectral data.

An electrical impedance study was performed to investigate the conducting nature of the GNB-modified-CF electrode. Nyquist and Bode plots obtained for bare CF and GNB-modified-CF electrodes in a K_3_[Fe(CN)_6_] (2.0 mM) redox couple containing ABS (pH 4.0 ± 0.05) at scanning frequencies from 0.01 to 100 000 Hz are shown in Fig. 6A–C. The electrode resistance (*R*_A_), electrolyte resistance (*R*_B_), and the diffusion layer resistance were calculated from Nyquist plots.^47^ The charge-transfer resistance (*R*_CT_) of the GNB-modified-CF electrode was calculated from the semicircle diameter at the higher frequencies appearing in the Nyquist plot, which is higher than the CF, and is due to the decrease in the access of the electrolyte redox couple to the nanobuds on the GNB-modified-CF electrode surface (Fig. 6A).^48,49^ This validates the important role of GNB on the electrode in reducing the surface resistance, which increases the conductance, and thereby provides a more effective platform for the development of the sensor.^50^

The obtained Nyquist plot of the GNB-modified-CF electrode showed evidence of the following two characteristics: (i) slope of *Z*_im_*versus Z*_re_ is unity, *i.e.*, an almost straight line,^51^ and (ii) a diffusion-controlled electrode process occurred.^52^Fig. 6B reveals that the Bode angles for bare CF (55°) and GNB-modified-CF (69°) electrodes are less than 90°, which suggests that these electrodes were not behaving like an ideal capacitor.^32^ Electrode electrical conductivities (*σ*_e_) of CF and the GNB-modified-CF electrode were calculated to be 9.00 × 10^−5^ and 1.38 × 10^−4^ S m^−1^, corresponding to electrode resistances of 111 and 72 Ω m^2^, respectively.^53^ From both the charge-transfer resistance and phase angle values, it was confirmed that the GNB-modified-CF electrode is more conductive than bare CF. At lower frequencies (−1.0 to 0.0 log Hz), the impedance (|*Z*|) value for the GNB-modified-CF electrode (72 Ω) is less than that of bare CF (111 Ω), but almost equal at 0.0 to 1.5 log Hz, and decreased at higher frequencies (1.5 to 5.0 log Hz)^32^ (Fig. 6C).

The simplified Randles equivalent circuit for the impedance spectroscopy measurements (Fig. 6C inset) and the obtained *R*_S_, *C*, and *R*_CT_ values are listed in Table SI1.† The calculated electrode conductivity and resistance using Nyquist plot values are well in harmony with the data obtained from the Randles equivalent circuit. From the impedance spectroscopy results, we concluded that our GNB-modified-CF electrode possesses lower electrode resistance and fast electron transfer when compared to the bare CF.

The effect of interferons on the GNB-modified-CF electrode's selectivity towards Ce^3+^ was examined in the presence of various metals ions with +3 oxidation state (Al^3+^, Cr^3+^, Dy^3+^, Fe^3+^, Gd^3+^, La^3+^, Pr^3+^, Ru^3+^, V^3+^, and Yt^3+^). From the obtained CV results, the relative error (RE) was calculated as the relative deviation in the peak current before and after the addition of interferons using eqn (1). The calculated RE was less than 10% for all the metal ions except for La^3+^, Ru^3+^, and V^3+^, (Table 1), which indicates that the other above mentioned metal ions did not affect the adsorbed Ce^3+^ on the working electrode surface. All the employed metal ions are electroactive, which can cause changes in the current. The equation to determine RE is as follows:1
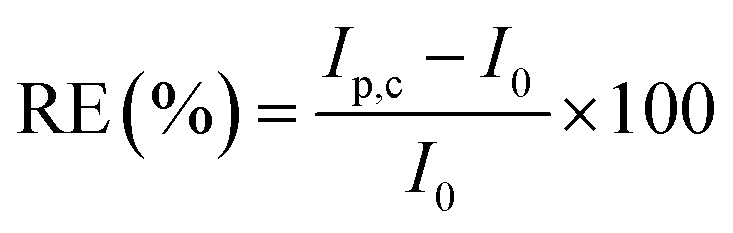
where *I*_p,c_, and *I*_0_ denote the peak current of the GNB-modified-CF electrode Ce^3+^ measured with and without interferon, respectively.

**Table 1:** Effect of interferons on the GNB-modified-CF electrode after Ce^3+^ adsorption

Sample no.	Interferon	*I*_p_	RE (%)
1	Al^3+^	6.474	5.148611
2	Ce^3+^	6.653	8.055871
3	Dy^3+^	6.655	8.088355
4	Fe^3+^	6.55	6.382979
5	Gd^3+^	6.569	6.691571
6	La^3+^	6.78	10.11856
7	Pr^3+^	6.634	7.74728
8	Ru^3+^	6.885	11.82394
9	V^3+^	7.316	18.8241
10	Yt^3+^	6.728	9.273997

**Fig. 7:**
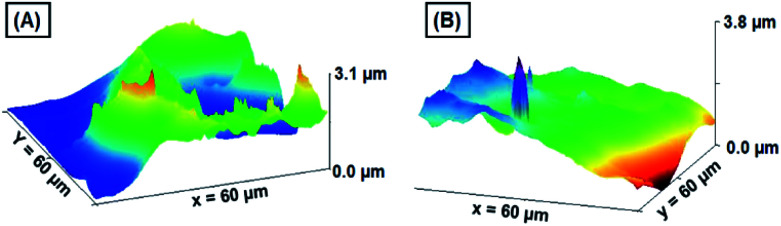
AFM images of the GNB-modified-ITO electrode (A) before and (B) after the determination of Ce^3+^ by CV analysis in ABS (pH 4.0 ± 0.05) at a scan rate of 50 mV s^−1^. Amplitude, spatial, and hybrid details can be found in Fig. SI9.†

Investigation of surface profile, roughness, and critical dimensions of the working electrode is obligatory for electrodes used in the electrochemical analysis. The average thickness of the GNB-modified-CF electrode before and after the electrochemical determination of Ce^3+^ was analyzed through a profilometer. Fig. SI8† shows 3D images of the profilometer, histogram, section analysis, and its corresponding graph. The mean thickness calculated for the GNB-modified-CF electrode after the electrochemical determination was approximately two times higher than that before the electrochemical experiment (33.11 → 62.67 μm), which is not a precise interpretation. We also experienced a bit of difficulty with the atomic force microscopy technique (AFM) when examining the changes in the surface topography of the GNB-modified-CF electrode before and after the electrochemical determination of Ce^3+^. Nevertheless, for a better understanding of the changes in the surface topography of the working electrode, a colloidal solution of GNB was coated onto the indium tin oxide (ITO) surface, and AFM images were recorded for the GNB-modified-ITO electrode before and after the electrochemical determination of Ce^3+^ (Fig. 7).

The electrochemical determination of Ce^3+^ by the GNB-modified-ITO electrode under similar experimental conditions can be found in Fig. SI7.† The 3D images of the homogeneously coated GNB-modified-ITO show a thickness of 3.1 μm (Fig. 7A). After electrochemical determination of Ce^3+^ by CV studies, the thickness increased by approximately 0.7 μm (3.1 → 3.8 μm) (Fig. 7B), which confirms the adsorption of Ce^3+^ molecules onto the GNB-modified-ITO electrode. The calculated average roughness (*R*_a_, 0.332 → 0.158 μm) and root mean square roughness (*R*_q_, 0.367 → 0.182 μm) of the working electrode decreased after electrochemical determination of Ce^3+^ (Fig. SI9†).^54^ Based on both profilometer and AFM results, we primarily concluded that the Ce^3+^ ions are adsorbed on the GNB-modified-CF electrode surfaces, and the nanobud morphology of the electrode material might not have changed even after the electrochemical experiments.

**Fig. 8:**
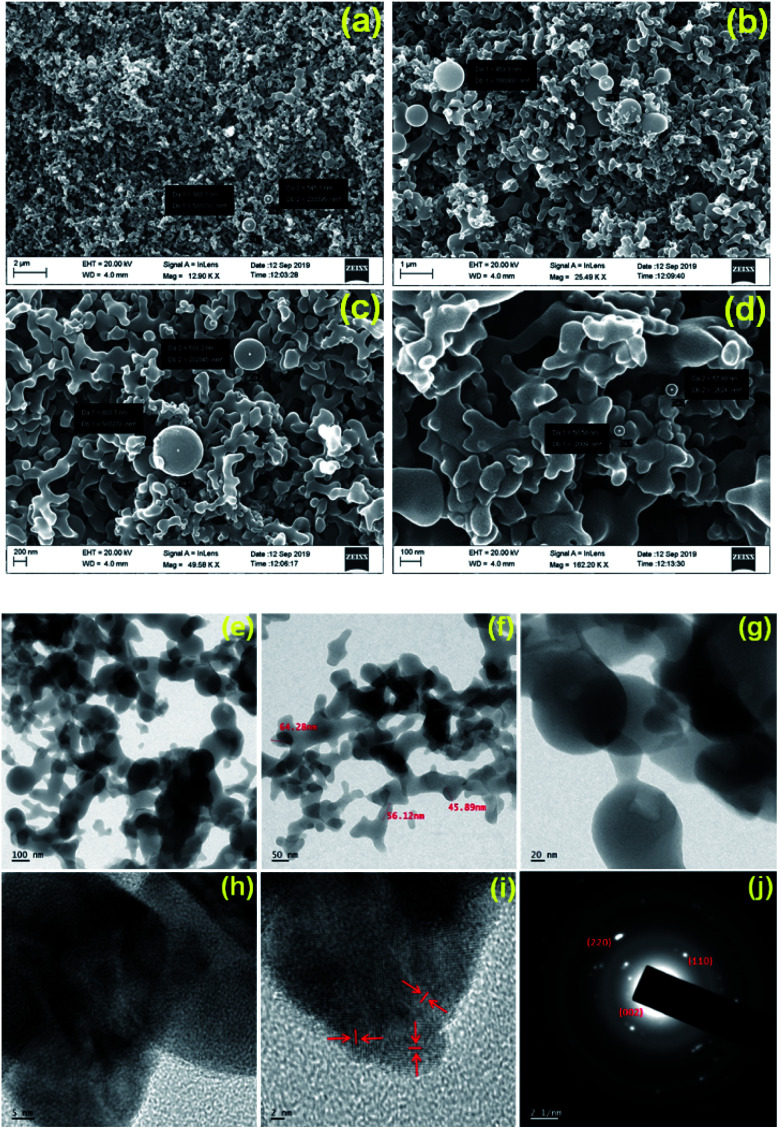
(a–d) FE-SEM and (e–j) HR-TEM images of the GNB-modified-CF electrode after the determination of Ce^3+^ by CV analysis in ABS (pH 4.0 ± 0.05) at a scan rate of 50 mV s^−1^. Scale bar (a–d) 2.0 μm, 1.0 μm, 200 nm, and 100 nm, respectively; scale bar (e–i) 100, 50, 20, 5 and 2 nm, respectively. Particle size distribution as a histogram can be found in Fig. SI11.† (j) SAED pattern. FE-SEM and HR-TEM images of GNB can be found in Fig. SI11a–c and SI11d–f,† respectively.

To predominantly corroborate the changes in the surface morphology of the GNB and to authenticate the adsorption of Ce^3+^ onto the surface of the GNB-modified-CF electrode, we recorded field emission-scanning electron microscopy (FE-SEM) and high-resolution-transmission electron microscopy (HR-TEM) images of the GNB-modified-CF electrode after the electrochemical determination of Ce^3+^. As illustrated in FE-SEM images, the morphology of the electrode material, DAN-GQD, remained the same as that of the nanobuds even after the electrochemical studies, but the average size of GNB marginally increased (35–40 → 50 nm) (Fig. 8a and d). This slight increase in average size (approximately 15 nm) could possibly be due to the decrease in the interlayer spacing, where Ce^3+^ acts as a connecting site. Furthermore, the internal strain caused by Ce^3+^ binding results in the unification of the nanobuds, thereby increasing their size.^55^ Interestingly, the diffusion induced during the electrochemical reaction can also cause fusion between neighboring nanobuds, which may be the potential reason for their increased size.^56,57^

The adsorption of Ce^3+^ onto the surface of the electrode was confirmed by EDS analysis and a corresponding mapping technique (Fig. SI10B†). The EDS data (Fig. SI10A†) indicates that among the elements present on the electrode surface, the 4.76 wt% corresponds to Ce^3+^, which concurs with the elemental mapping data. The HR-TEM images also confirm the slight increase in the average size of GNB (35–40 → 55 nm) upon Ce^3+^ sensing without changing its nanobud morphology (Fig. 8e–h). The selected area electron diffraction (SAED) pattern demonstrates the crystalline nature of Ce^3+^ ion with Debye–Scherrer diffraction corresponding to the (110) and (220) planes of Ce^3+^ and a graphitic plane (002)^58^ (inset Fig. 8j). Additionally, the observed ring pattern of the reflected planes from SAED is attributed to the fluorite structure of CeO_2_.^59,60^ It is noteworthy to mention that different patterns of fringes observed in high-resolution images of the DAN-GQD·Ce^3+^complex are attributed to different planes of Ce^3+^ (Fig. 8i),^61^ which was not witnessed in the SAED of free GNB (Fig. SI11†).^25^ From the above-mentioned electron microscopy results, we largely concluded that DAN-GQD in nanobud morphology can be propitious for the electrochemical determination of Ce^3+^ ions without changing its morphology even in acidic conditions.

**Fig. 9:**
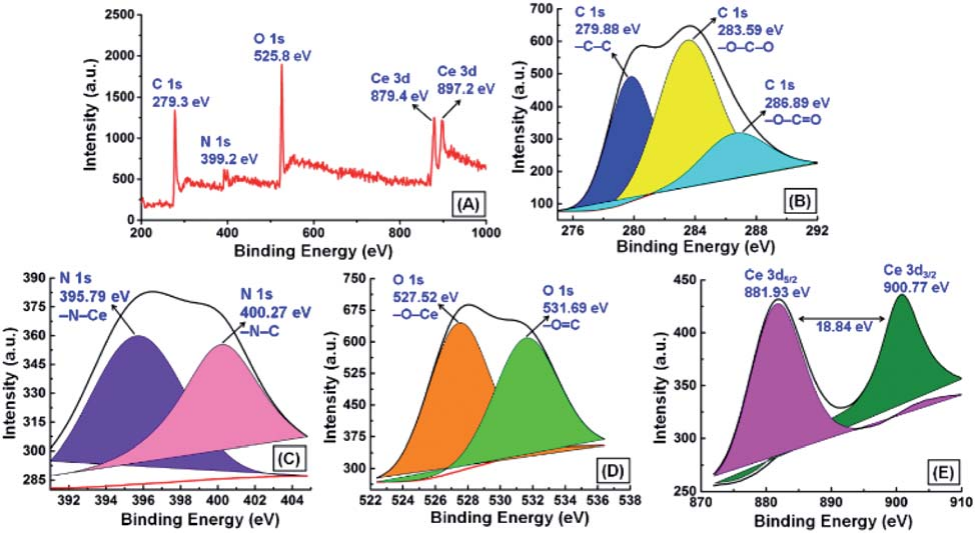
X-ray photoelectron spectrum of the GNB-modified-CF electrode after the determination of Ce^3+^ by CV analysis in ABS (pH 4.0 ± 0.05) at a scan rate of 50 mV s^−1^: (A) survey spectrum; high-resolution XPS of (B) C 1s, (C) N 1s, (D) O 1s, and (E) Ce 3d. The XPS spectrum of DAN-GQD can be found in Fig. SI13.†

To distinguish different chemical states of components, chemical composition, and oxidation state of the adsorbed Ce^3+^, X-ray photoelectron spectroscopy (XPS) analysis was carried out for the GNB-modified-CF electrode after the electrochemical determination of Ce^3+^. The XPS survey spectrum of the GNB-modified-CF electrode indicates the presence of C 1s at 279.3 eV, N 1s at 399.2, O 1s at 525.8 eV, and Ce 3d at 879.4 and 897.2 eV (Fig. 9A), and with C, N, O, and Ce content of 67.25, 10.97, 18.97, and 2.82 at% (Fig. SI12A†), respectively,^62^ which is in accord with the energy dispersive X-ray analysis (EDAX) data and the XPS survey spectrum of the GNB-modified-ITO electrode (Fig. SI12B†). The high-resolution C 1s spectrum shows distinct –C–C, –O–C–O, and –O–C=O peaks at 279.9, 283.6, and 286.9 eV, respectively, which divulges that the electrode material retained its graphitic-like structure even after the electrochemical studies (Fig. 9B).^63^ In the high-resolution N 1s spectrum, two distinct peaks were observed at 395.8 and 400.3 eV that were attributed to –N–Ce and –N–C, respectively (Fig. 9C). The sharp peak perceived at 395.8 eV ostensibly denotes that the nature of the nitrogen bonded to the Ce^3+^ is of the primary amine (–NH_2_) category, which is approximately 2.3 eV less than free –NH_2_ existing in DAN-GQD (398.1 eV) (Fig. SI13C†). The peak appearing at approximately 400 eV confirmed the non-participation of the secondary amine (–HN–C) present in the GNB during the complexation with Ce^3+^.

The high-resolution O 1s spectrum exhibited two peaks at 527.5 and at 531.7 eV, which were attributed to the –O–Ce and –O=C and indicate the involvement of oxygen atoms in the complexation (Fig. 9D). The high-resolution spectrum of Ce 3d shows two well-separated peaks at 881.9 and 900.8 eV, which resulted from the partaking of two spin-orbitals 3d_5/2_ and 3d_3/2_, respectively, with split-orbital separation of approximately 18.8 eV (Fig. 9E).^64^ The peak at approximately 900 eV indicated that the adsorbed Ce^3+^ on the modified electrode bonds with additional oxygen atoms, for instance, the oxygen of the hydroxyl and carboxyl groups present in the GNB, which is in accordance with already reported CeO_2_ compounds.^65,66^ The peak observed at approximately 882 and 901 eV in both surveys and the high-resolution spectrum of Ce 3d indicates that the cerium ions adsorbed onto the modified electrode surface are in a +4 oxidation state, not a +3 state.^67^ Based on the obtained XPS results, we strongly concluded that the formation of the DAN-GQD·Ce^3+^ complex could occur through the participation of the oxygen of hydroxyl and carboxyl groups and nitrogen of primary amine, which might be thoroughly confirmed by X-ray diffraction studies and theoretical studies.

**Fig. 10:**
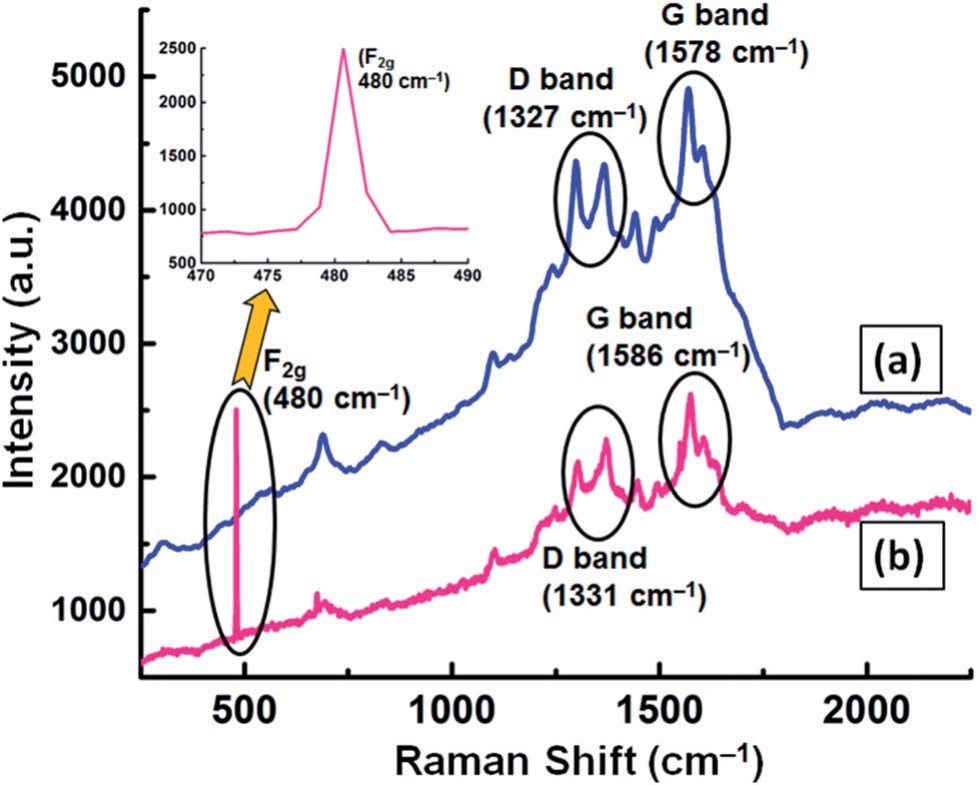
Micro Raman spectrum of the GNB-modified-CF electrode (a) before and (b) after the electrochemical determination of Ce^3+^ by CV analysis in ABS (pH 4.0 ± 0.05) at a scan rate of 50 mV s^−1^. The inset shows the existence of a Raman active F_2g_ peak at 480 cm^−1^, which corresponds to the presence of fluorite-type Ce^3+^ on the electrode surface. The Raman spectrum of GNB can be found in Fig. SI14.†

Micro Raman spectroscopy analysis was carried out for the GNB-modified-CF electrode before and after the electrochemical sensing experiment to understand the defects present in the graphene moiety. Before the sensing experiment, the modified electrode exhibited D and G bands centered at approximately 1327 and 1578 cm^−1^, respectively, with an intensity ratio (*I*_D_/*I*_G_) of 0.927 (Fig. 10a). However, after the sensing experiment, the D and G bands showed a slight blueshift (1331 and 1586 cm^−1^, respectively) with an intensity ratio (*I*_D_/*I*_G_) of 0.894 (Fig. 10b). This very negligible *I*_D_/*I*_G_ ratio difference (0.033) confirmed that the graphitic nature of the GNB-modified-CF electrode did not change even after the electrochemical experiment.^68^ Furthermore, the Raman active F_2g_ peak observed at approximately 480 cm^−1^ was evidence for the binding of fluorite-type Ce^3+^ ions with oxygen groups available in the GNB^69,70^ (Fig. 10 inset). The obtained micro Raman results are well in accordance with the FE-SEM and HR-TEM results proving that the Ce^3+^ ions are adsorbed on the modified electrode surface without causing any defects on the electrode material.^71^

**Fig. 11:**
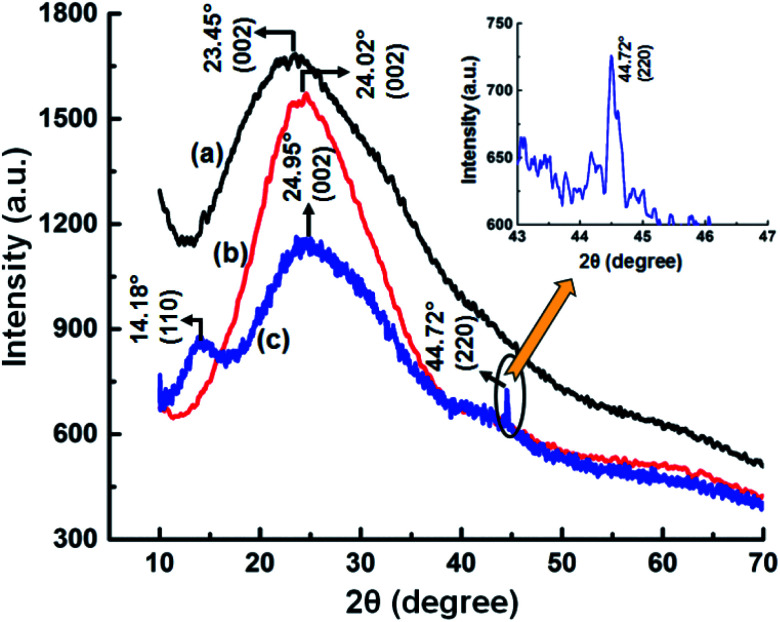
X-ray diffraction profiles of (a) GQD, (b) GNB, and (c) the GNB-modified-CF electrode after the Ce^3+^ ion electrochemical sensing studies (inset). Existence of a peak at 44.72° (220 planes), which corresponds to the presence of a fluorite-type crystal structure of Ce on the electrode surface.

To further confirm the adsorption of Ce^3+^ ion on the GNB-modified-CF electrode surface, we carried out X-ray diffraction studies for the GNB-modified-CF electrode after the electrochemical studies, and the results were compared with those for GQDs and GNB (Fig. 11). GQDs and the electrode material, GNB, exhibited 2*θ* broad diffraction peaks centered at 23.45° and 24.02°, respectively, which were attributed to the (002) plane of the graphitic structure (Fig. 11a and b). After the electrochemical studies, the X-ray diffraction (XRD) profile of the GNB-modified-CF electrode was centered at 24.95° (002), which indicates that there was no change in the graphitic structure of GNB after the adsorption of Ce^3+^ ion onto the electrode surface^25^ (Fig. 11c). Moreover, upon determination, the presence of Ce^3+^ ion created internal strain in GNB and, as a result, shifted the 2*θ* towards a higher value (24.02° → 24.95°) and diminished the peak intensity (1555 → 1140 atomic units (a.u.)).^72^ There was also a decrease in the peak intensity of the GNB-modified-CF electrode due to the increased size of the electrode material (approximately 50 nm) when compared with GNB (approximately 35–40 nm), which is in analogy with the FE-SEM results.

The adsorption of Ce^3+^ ion on the GNB-modified-CF electrode surface was positively confirmed by the presence of two peaks corresponding to the (110) and (220) planes at 14.18° and 44.72°, respectively (JCPDS 86-2451).^73,74^ The existence of a peak at 44.72° (Fig. 11 inset) indicates the crystallinity of Ce present on the electrode surface, which is similar to the fluorite-type crystal structure of Ce. This Ce presence may be due to the formation of a bond between Ce^3+^ and the deprotonated –OH functional group of GNB.^75^ It is noteworthy to mention that the peak at 14.18° materializes out only when Ce^3+^ ion is attached to the nitrogen atom of the primary amine moiety (–NH_2_) present in the GNB.^73^ Interlayer *d* spacing for the GNB-modified-CF electrode before and after the adsorption of Ce^3+^ was calculated to be approximately 3.701 and 3.564 Å, respectively. From the XRD results, we were able to strongly conclude that the (i) adsorption of Ce^3+^ on the GNB-modified-CF electrode surface and (ii) the determination of Ce^3+^ by GNB were successful.

## Conclusions

**Scheme 1:**
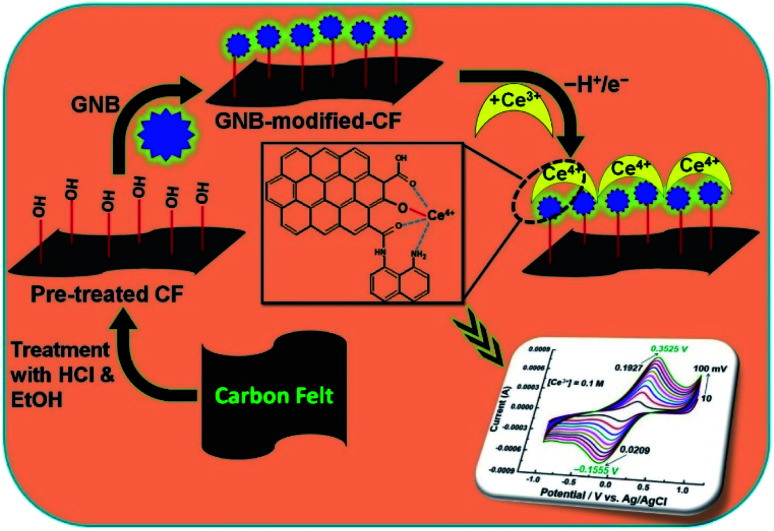
Representation of plausible Ce^3+^ adsorption on a GNB-modified-CF electrode.

The working electrode, a GNB-modified-CF electrode, was prepared by a simple drop-coating method for the sensitive and selective detection of Ce^3+^ in ABS (pH 4.0 ± 0.05). The GNB-modified-CF electrode exhibited excellent stability and electrochemical activity towards Ce^3+^ at different scan rates (10 → 100 mV s^−1^), in various concentrations of Ce^3+^ (0.10 M; 0 → 200 μL), and at different pH values (3 → 12). The impedance data reveal that the GNB-modified-CF electrode is not an ideal capacitor, but the electron transfer was more favorable at the GNB-modified-CF electrode than the bare CF. The developed GNB-modified-CF electrode is extremely stable and reproducible toward Ce^3+^ (Scheme 1).

AFM images, XPS, Raman, and XRD analyses of the working electrode confirmed the adsorption of Ce^3+^ onto the electrode surface. FE-SEM and HR-TEM images of the working electrode obtained after Ce^3+^ ion adsorption markedly indicate that the size of GNB slightly increased (from 35–40 to 50–55 nm) without a change in its morphology (nanobuds), which is in concurrence with the XRD results. Moreover, from all the electrochemical experiments, the obtained limit of detection (approximately 2.60 μM) was adequately low for the detection of submillimolar concentration ranges of Ce^3+^ in numerous chemical and biological systems.

After a thorough literature survey, we confirmed that the current report is the firm report for the determination of Ce^3+^ by functionalized graphene quantum dots (DAN-GQDs) with a nanobud morphology (NB) using electrochemical methods (CV and DPV) within a much less than adequate Ce^3+^ concentration (Table SI2†). Further studies on the determination of other biologically and environmentally important metal ions are currently in progress.

## Experimental

### Materials and methods

All the reagents and solvents involved in synthesis were analytical grade and used without any further purification. Citric acid, K_3_[Fe(CN)_6_], cerium(iii) nitrate, and ABS were purchased from Sigma-Aldrich, India. Electrochemical measurements were performed using a three-electrode system with thoroughly washed and dried DAN-GQD-coated CF as a working electrode, Pt wire as a counter electrode, and KCl-saturated Ag/AgCl as a reference electrode. Electrochemical studies were carried out using an Autolab electrochemical workstation (Metrohm, The Netherlands) in ABS (0.2 M; pH 4.0 ± 0.05). CF was purchased from Alfa Aesar, Chennai, India.

X-ray diffraction measurements were carried out using Cu Kα radiation as an X-ray source (*λ* = 1.542 Å) (X'pert PRO PANalytical instrument, The Netherlands). AFM images were recorded using APE Research, Italy, in non-contact mode. The surface morphology of the working electrode was analyzed by HRTEM (200 kV FE-TEM, model JEM-2100F, JEOL) at IIUCNN–Mahatma Gandhi University, Kottayam, India, and FE-SEM-EDAX (SIGMA HV–Carl Zeiss with Bruker Quantax 200–Z10 EDS Detector) at Coimbatore Institute of Technology, Coimbatore, India. Micro Raman spectral analysis was carried out using Horiba Lab Ram HR with a 532 nm laser, 1800 grating, at Bharathiar University, Coimbatore, India. X-ray photoelectron spectroscopy measurements were carried out using the synchrotron at RRCAT, Indore, India equipped with a double crystal monochromator (BL-14) and the energy source of a bending magnet of 15.0 keV. Thermal studies were carried out with a PerkinElmer Simultaneous Thermal Analyzer (STA6000) at the DRDO-BU Center for Life Sciences at Bharathiar University, Coimbatore, India. Profilometer images were recorded with a 3D Optical Profilometer Xeta20.

## Conflicts of interest

On behalf of all authors, the corresponding author declares that there are no conflicts of interest.

## Supplementary Material

RA-010-D0RA07555H-s001
