# Supplementary material for: Surface and morphology analyses, and voltammetry studies for electrochemical determination of cerium(iii) using a graphene nanobud-modified-carbon felt electrode in acidic buffer solution (pH 4.0 ± 0.05)
Source: RSC Adv. 2020 Oct 9;10(61):37409–18. doi: 10.1039/d0ra07555h (PMC9057166; doi:10.1039/d0ra07555h)
Supplement: RA-010-D0RA07555H-s001 [file RA-010-D0RA07555H-s001.pdf]

Electronic Supplementary Material (ESI) for RSC Advances.
This journal is © The Royal Society of Chemistry 2020

Supporting Information

Surface and Morphology analyses, and Voltammetry studies for electrochemical determination of Cerium(III) using Graphene Nanobud–modified–Carbon felt electrode in Acidic Buffer Solution (pH 4.0±0.05)

Pavithra V. Ravi,[1] Daniel T. Thangadurai,[1] Kasi Nehru,[2] Yong Ill Lee,[3] Devaraj Nataraj,[4] Sabu Thomas,[5] Nandakumar Kalarikkal,[5] and Jiya Jose[5]*

[1]Department of Nanotechnology, Sri Ramakrishana Engineering College, Affiliated to Anna University,Coimbatore–641022,Tamilnadu,India.
[2]Department of Chemistry, Anna University – Bharathidasan Institute of Technology, Tiruchirappalli–620024,India.
[3]Department of Chemistry, Changwon National University, Changwon 641-773, South Korea.
[4]Department of Physics, Bharathiar University, Coimbatore – 641046, Tamilnadu, India.
[5]International and Inter University Centre for Nanoscience and Nontechnology, Mahatma Gandhi University, Kottayam – 686560, Kerala, India.

1. Characterization of GNB

1.1. Thermal characterization

Examination of thermal stability of an electrode material is vital for electrochemical applications, which are in a straight line with device performance such as efficacy, lifespan, etc. Thermal stability of as-prepared GNB was examined by Thermo-Gravimetric analyses (TGA) and Differential Scanning Calorimetric (DSC) techniques in a heating range from 50 to 900 °C with a heating rate of 10 °C min^{-1} with N_2 flow of 20 mL min^{-1} (Figure SI1). The first weight loss up to 100 °C represents the evaporation of water molecule present in GNB. Continuous weight loss at 200–500 °C may be characteristic of loss of covalently attached oxygen-containing surface functional groups linked to the edges of the GNB.[1] The final deprivation above 500 °C shows the decomposition of the carbon backbone.[2] DSC analysis results imply a distinct exothermic peak accompanying a significant weight loss around 225 °C indicating that there is one chemical reaction involved in the process (inset, Figure SI1). The decomposition of carbon backbone of graphitic structure was observed above 500 °C, which is in well accord with the TGA analysis.

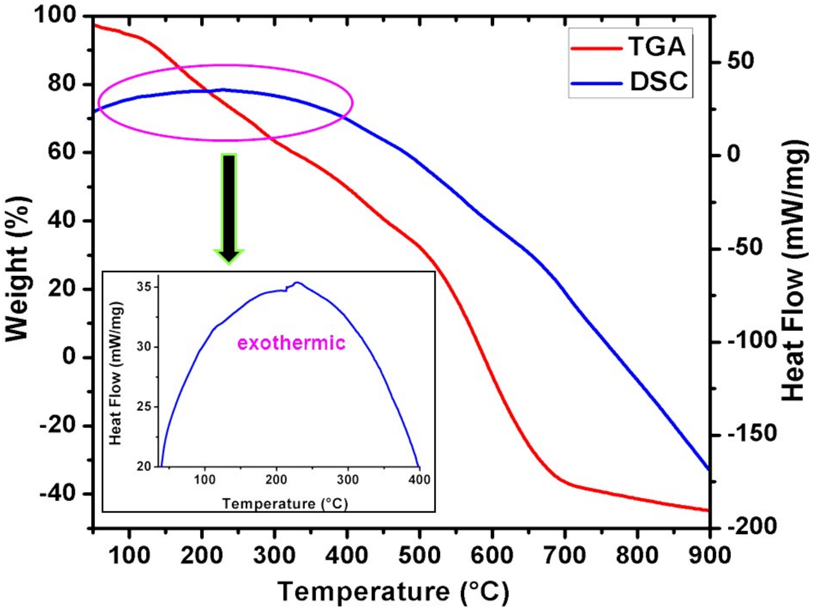

Figure SI1. Thermal analyzes of GNB in N_2 flow (20 mL/min) at a heating rate of 10 °C/min.

2. Fabrication of Working Electrode

The carbon felt pieces (1 cm × 1 cm) were dipped into 2.0 M of HCl solution (10 mL) for 10 min, followed by immersion in ethanol solution (2 mL) and then rinsed thoroughly with distilled water (2×10 mL). The resulting carbon felt was dried completely in room temperature. GNB (2.0 mg) colloidal solution was coated on the pretreated carbon felt and kept for drying in the incubator at 80 ºC for 12 h.

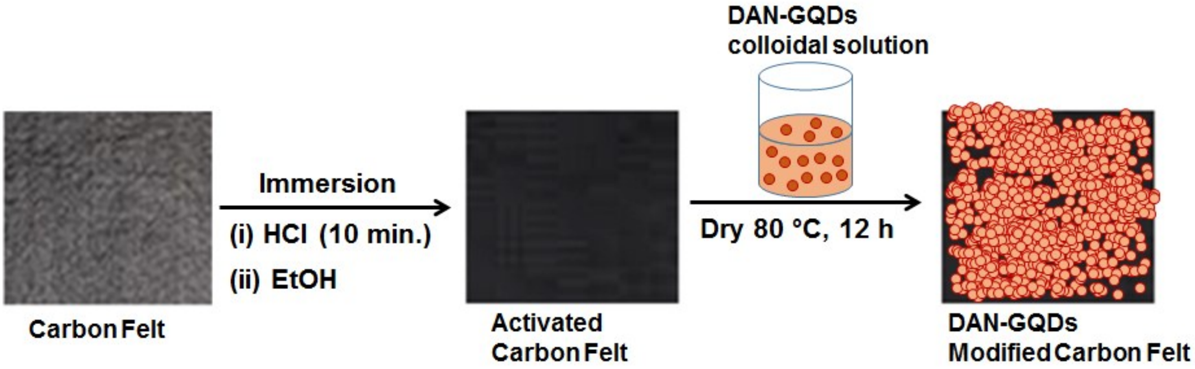

Figure SI2. Schematic illustration of fabrication of GNB–modified–CF electrode.

3. Electrochemical studies

3.1. CV and DPV studies

The electrochemical studies were performed with three electrode system; Ag/AgCl was used as reference electrode, Pt wire as counter electrode and GNB–modified–CF as working electrode

in Acidic Buffer Solution (pH 4.0±0.05). Initially, the CV (with scan rate of 50 mVs^{-1}) and DPV (with set potential of 0.25 V) studies were carried out with bare CF, followed with GNB–modified–CF electrode upon addition of Ce^{3+} (0.1 M). CV studies were also carried out with (i) fixed scan rate (50 mV/s) in different concentration of Ce^{3+} solution (0.1 M; 0→200 μL), (ii) fixed concentration of Ce^{3+} solution (0.1 M) in different scan rate (10→100 mV/s), and (iii) interferon studies. The influence of electrolyte pH (3→12) on the electrochemical behavior of GNB–modified–CF electrode upon addition of Ce^{3+} was carried out with scan rate of 50mVs^{-1} at fixed concentration of Ce^{3+}(0.1M). Impedance studies were carried out with $K_3[Fe(CN)_6]$ (2.0 mM) redox couple containing in Acidic Buffer Solution (pH 4.0±0.05) at scanning frequencies from 0.01 to 100,000 Hz. For every measurement, newly prepared GNB–modified–CF electrode was used as working electrode. Each experiment was repeated at least thrice till consistent values were obtained.

Linear Fit (17-05-2019 12:00:27)

Parameters

		Value	Standard Error
B	Intercept	2.37901E-4	5.74344E-6
	Slope	5.98677E-6	1.03909E-7

Statistics

	B
Number of Points	7
Degrees of Freedom	5
Residual Sum of Squares	0.17875
Pearson's r	0.99925
Adj. R-Square	0.9982

Summary

	Intercept		Slope		Statistics
	Value	Standard Error	Value	Standard Error	Adj. R-Square
B	2.37901E-4	5.74344E-6	5.98677E-6	1.03909E-7	0.9982

ANOVA

		DF	Sum of Squares	Mean Square	F Value	Prob>F
B	Model	1	118.67755	118.67755	3319.56499	2.97989E-8
	Error	5	0.17875	0.03575		
	Total	6	118.8563			

Figure SI3. Linear fit parameters for the calculated LoD from the CV obtained for GNB–modified–CF electrode with Ce^{3+} (0.1 M; 10 μL) in ABS (pH 4.0±0.05) at different scan rate (10-100 mV/s).

Linear Fit (17-05-2019 13:01:52)

Parameters

		Value	Standard Error
B	Intercept	5.48716E-6	3.26912E-7
	Slope	3.98356E-7	5.92321E-9

Statistics

	B
Number of Points	8
Degrees of Freedom	6
Residual Sum of Squares	0.4031
Pearson's r	0.99934
Adj. R-Square	0.99845

Summary

	Intercept		Slope		Statistics
	Value	Standard Error	Value	Standard Error	Adj. R-Square
B	5.48716E-6	3.26912E-7	3.98356E-7	5.92321E-9	0.99845

ANOVA

		DF	Sum of Squares	Mean Square	F Value	Prob>F
B	Model	1	303.87181	303.87181	4523.00836	7.2696E-10
	Error	6	0.4031	0.06718		
	Total	7	304.27491			

Figure SI4. Linear fit parameters for the calculated LoD from the CV obtained for GNB–modified–CF electrode with Ce^{3+} (0.1 M; 0→200 μL) in ABS (pH 4.0±0.05) at a scan rate of 50 mV/s.

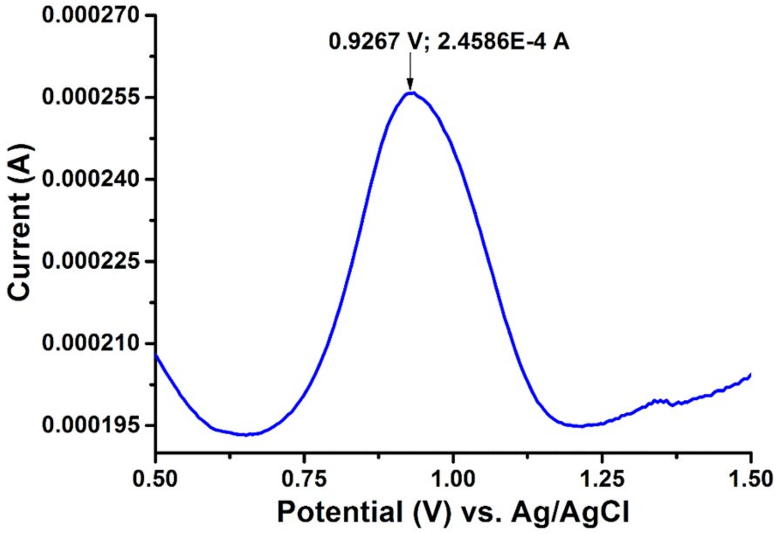

Figure SI5. DPV obtained for CF with Ce^{3+} (0.1 M; 10 μL) in ABS (pH 4.0±0.05) at a scan rate of 10 mV/s.

Linear Fit (16-05-2019 22:45:46)

Parameters

		Value	Standard Error
B	Intercept	5.19768E-5	1.90375E-6
	Slope	2.19863E-6	4.06291E-8

Statistics

	B
Number of Points	12
Degrees of Freedom	10
Residual Sum of Squares	22.75935
Adj. R-Square	0.99152

Summary

	Intercept		Slope		Statistics
	Value	Error	Value	Error	Adj. R-Square
B	5.19768E-5	1.90375E-6	2.19863E-6	4.06291E-8	0.99152

ANOVA

		DF	Sum of Squares	Mean Square	F Value	Prob>F
B	Model	1	2928.38653	2928.38653	1286.67407	6.73517E-12
	Error	10	22.75935	2.27593		
	Total	11	2951.14588			

Figure SI6. Linear fit parameters for the calculated LoD from the DPV obtained for GNB–modified–CF electrode with Ce^{3+} (0.1 M; 0→200 μL) in ABS (pH 4.0±0.05) at a scan rate of 10 mV/s.

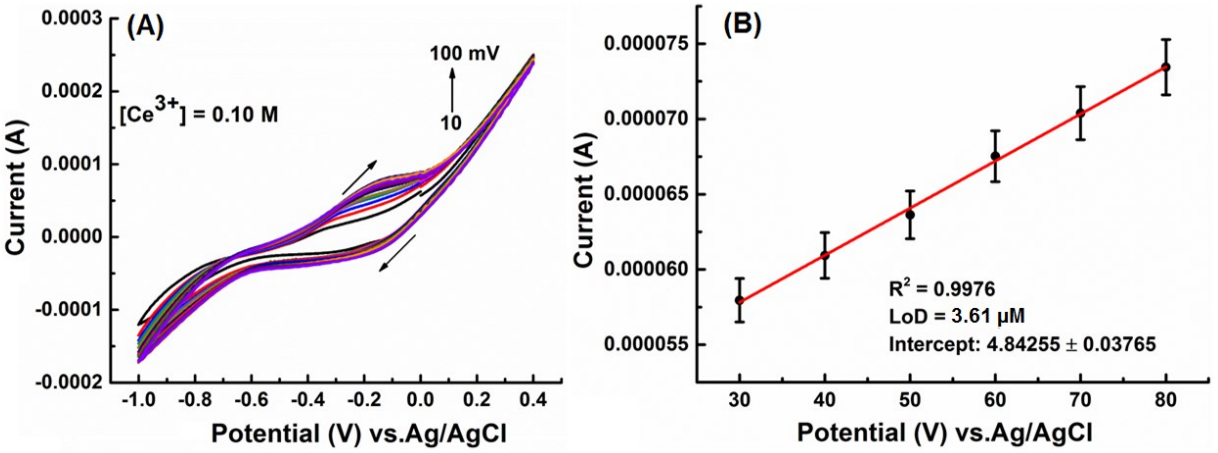

(C)

Linear Fit (26-09-2019 10:38:45)

Parameters

		Value	Standard Error
B	Intercept	4.84255E-5	3.76516E-7
	Slope	3.13015E-7	6.85851E-9

Statistics

	B
Number of Points	6
Degrees of Freedom	4
Residual Sum of Squares	0.12302
Pearson's r	0.99904
Adj. R-Square	0.9976

Summary

	Intercept		Slope		Statistics
	Value	Standard Error	Value	Standard Error	Adj. R-Square
B	4.84255E-5	3.76516E-7	3.13015E-7	6.85851E-9	0.9976

ANOVA

		DF	Sum of Squares	Mean Square	F Value	Prob>F
B	Model	1	64.05905	64.05905	2082.91407	1.37854E-6
	Error	4	0.12302	0.03075		
	Total	5	64.18207			

Figure SI7. (A) CV obtained for GNB–modified–ITO electrode with Ce^{3+} (0.1 M; 10 µL) in ABS (pH 4.0±0.05) at different scan rate (10-100 mV/s); (B) Influence of scan rate on reduction peak current. Error bars = standard deviation of three independent measurements made from newly prepared GNB–modified–ITO electrode; (C) Linear fit parameters for the calculated LoD.

From the linear fit plot, slope and intercept is calculated. LOD is obtained from the slope and intercept by using the following equation:

$$\text{LoD} = [3\times(\text{intercept/slope})]$$

3.2. Impedance spectroscopy measurements

The electrical resistance (R_A) (in Ω m^2) per unit square area of the planar electrode can be expressed as $R_A = L_e/\sigma_e$ where, L_e = electrode thickness, σ_e= electrode conductivity.[3] Using the above equation the conductance of the CF and GNB–modified–CF electrode were calculated. From the charge-transfer resistance, phase angle values, Nyquist plot and Randles circuit, it is confirmed that the GNB–modified–CF electrode is more conductive than the bare CF.

Table SI1. The obtained parameter values from the impedance spectra circuit for CF and GNB–modified–CF electrode in Acidic Buffer Solution (pH 4.0±0.05).

S.No	Element	Parameter	CF		DAN-GQDs-modified-CF	
			Value	Error %	Value	Error %
1	R_s	R	114.23	1.078	72.903	1.854
2	R_p	R	4180.7	2.775	68192	28.611
3	CPE	Y0	9.27×10^{-5}	3.237	8.73×10^{-5}	3.22
4	N		0.75834	0.989	0.79824	1.054
5	γ^2		1.0367		3.0777	
			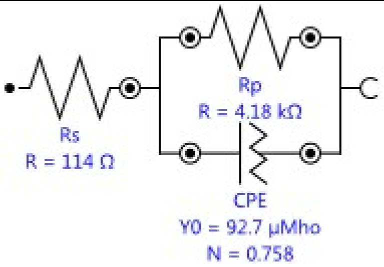		For circuit, please refer Figure 7 in main text.	

Where, Rs – Resistor in series (electrode resistance) ; Rp – Resistor in parallel (electrolyte resistance); CPE – Constant Phase element; N – phase change value occurred while fitting circuit; γ^2 – Chi-Squared.

4. Analysis of GNB–modified–CF electrode surface

(A)

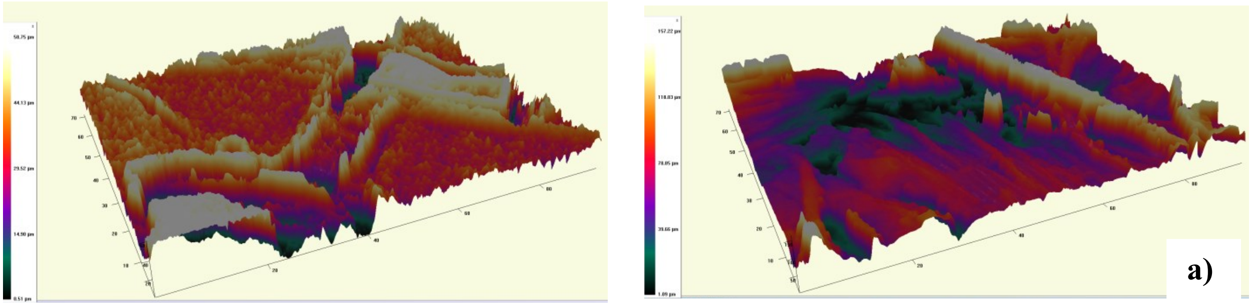

a)

(B)

Zeta Analysis Report
Image Name: Zeta Image3 File Name:
Date Acquired: Fri Aug 02 15:01:06 2019 Today:Fri Aug 02 15:01:06 2019
Z Range: 61µm No. of Steps: 200 Step Size: 0.308µm Field of View: 95µm x 71µm

	Cursor Left		Cursor Right		Cursor L-R		
	Avg Ht	Width	Avg Ht	Width	Step	Dist	Angle
1	34.16	7.990	25.32	7.904	-8.843	83.63	-6.036
2	36.97	7.990	32.13	7.904	-4.845	83.63	-3.315
3	36.67	7.990	35.15	7.904	-1.511	83.63	-1.035
Min	34.16	7.990	25.32	7.904	-8.843	83.63	-6.036
Max	36.97	7.990	35.15	7.904	-1.511	83.63	-1.035
Mean	35.93	7.990	30.87	7.904	-5.066	83.63	-3.462
SD	1.257	0	4.112	0	2.997	0	2.044
Var%	3.50%	0%	13.3%	0%	-59.2%	0%	-59.0%

a)

Zeta Analysis Report
Image Name: Zeta Image6 File Name:
Date Acquired: Fri Aug 02 15:09:18 2019 Today:Fri Aug 02 15:09:19 2019
Z Range: 163µm No. of Steps: 200 Step Size: 0.820µm Field of View: 95µm x 71µm

	Cursor Left		Cursor Right		Cursor L-R		
	Avg Ht	Width	Avg Ht	Width	Step	Dist	Angle
1	46.51	7.990	55.31	7.904	8.803	83.63	6.009
2	56.00	7.990	50.96	7.904	-5.044	83.63	-3.452
3	68.15	7.990	99.13	7.904	30.98	83.63	20.32
Min	46.51	7.990	50.96	7.904	-5.044	83.63	-3.452
Max	68.15	7.990	99.13	7.904	30.98	83.63	20.32
Mean	56.89	7.990	68.46	7.904	11.58	83.63	7.627
SD	8.855	0	21.75	0	14.84	0	9.774
Var%	15.6%	0%	31.8%	0%	128.1%	0%	128.1%

b)

(C)

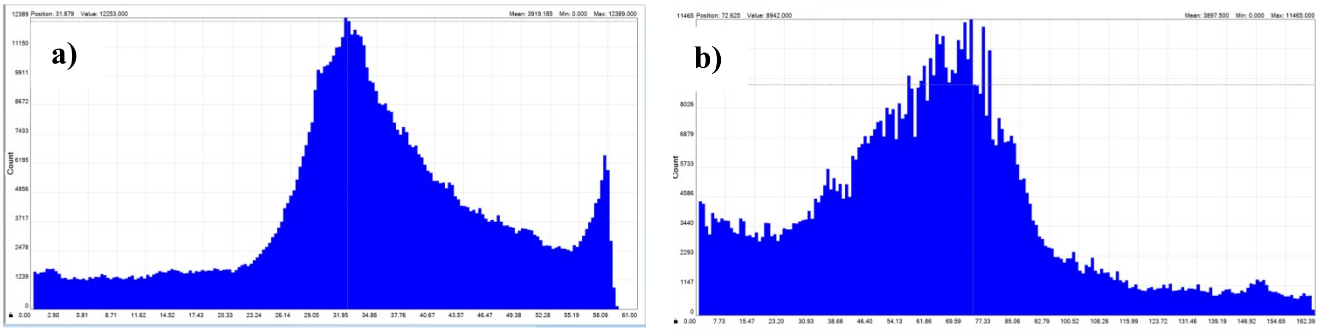

(D)

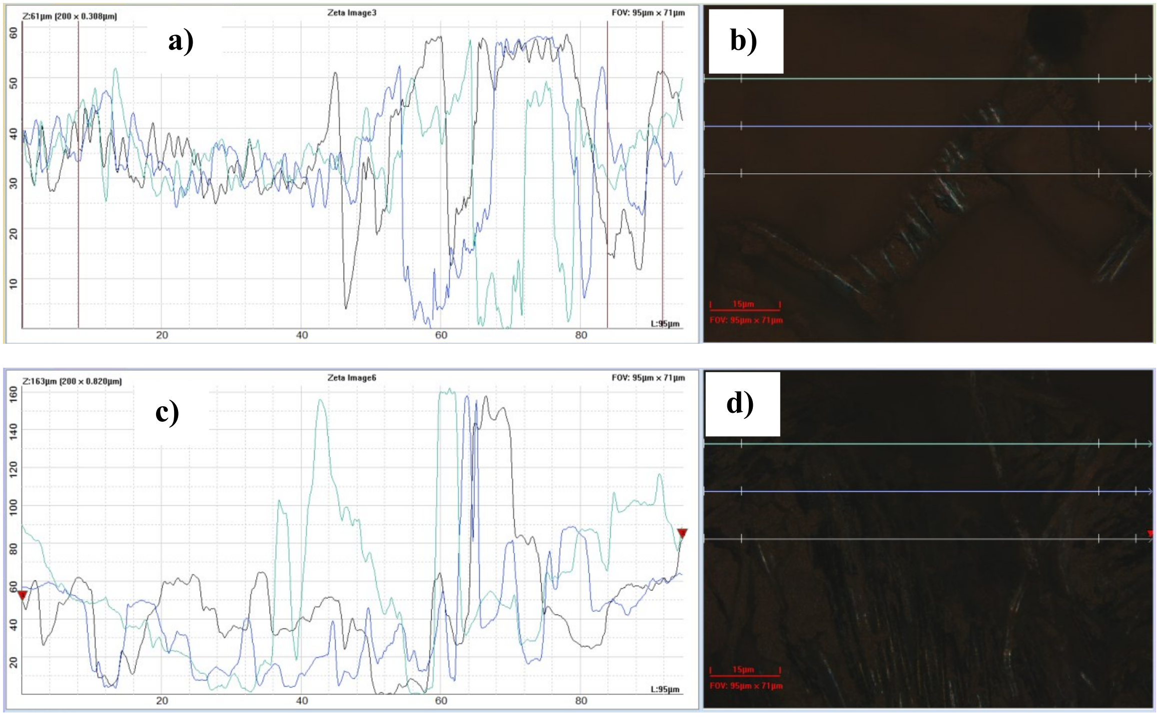

Figure SI8. (A) Optical profilometer 3D images of GNB–modified–CF electrode before (a) and after (b) electrochemical determination. (B) Corresponding surface parameter details, (C)

histogram of surface parameter, and (D) section analysis of the working electrode before (a and b) and after (c and d) the electrochemical determination.

(A)

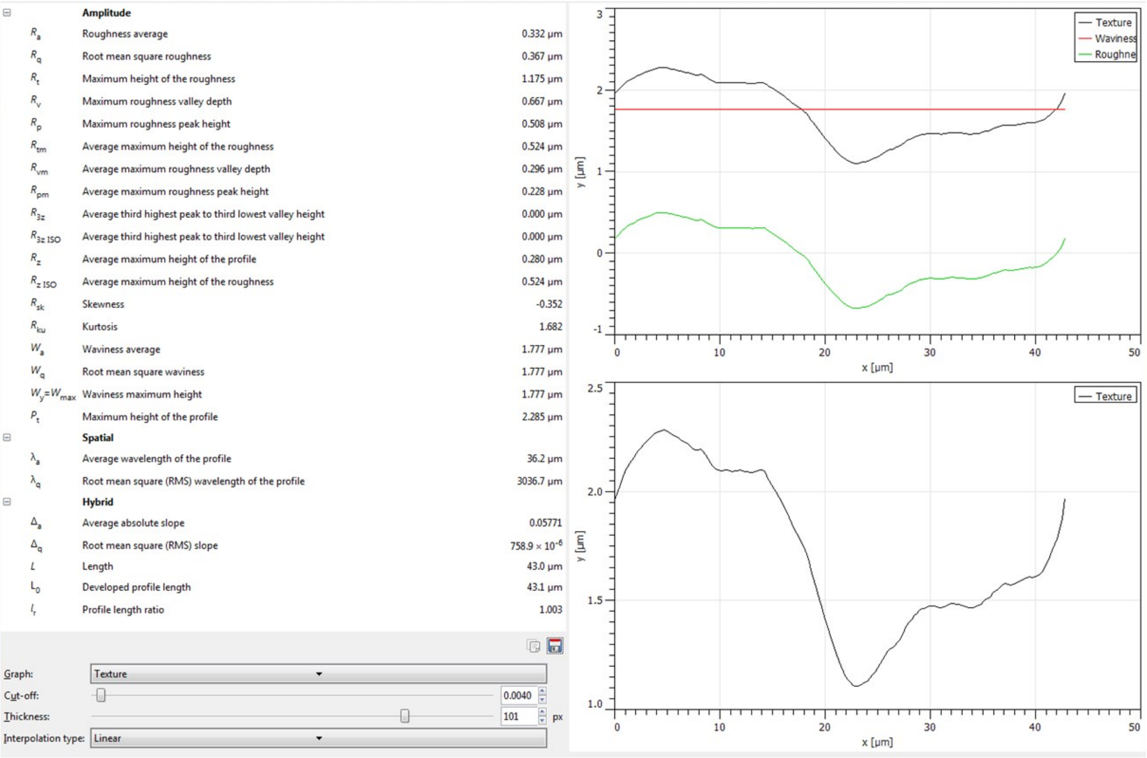

(B)

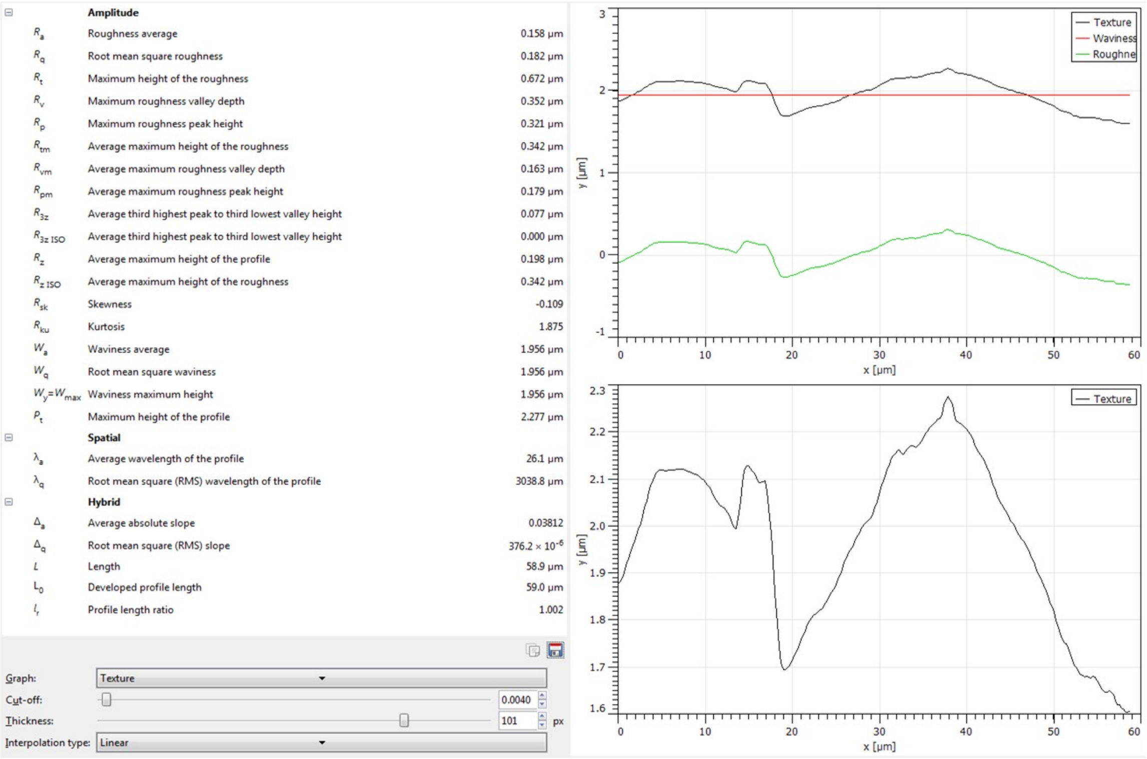

Figure SI9. Amplitude, spatial and hybrid details of AFM images of GNB-modified-ITO electrode (A) before and (B) after the determination of Ce^{3+} by CV analysis.

(A)

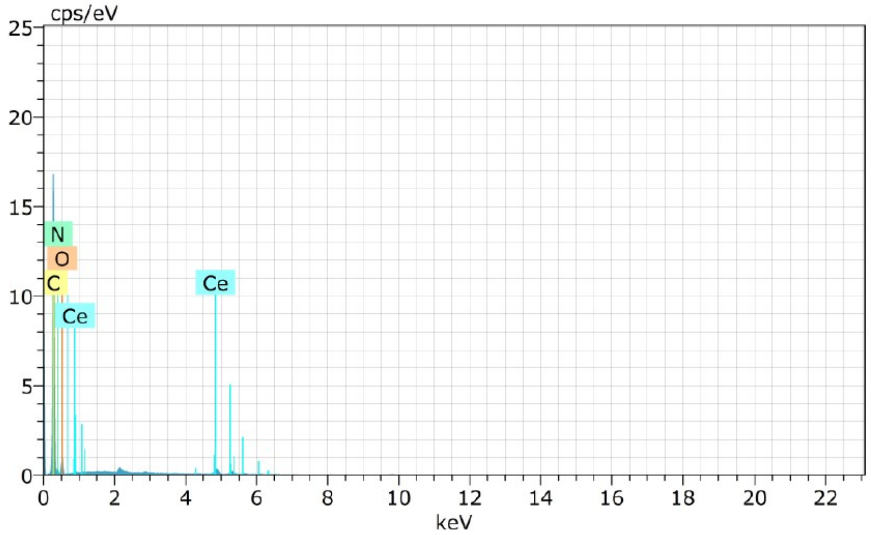

Spectrum: Objects 5008

El	AN	Series	unn. C [wt.%]	norm. C [wt.%]	Atom. C [at.%]	Error (1 Sigma) [wt.%]
C	6	K-series	66.85	66.85	74.23	7.51
O	8	K-series	14.37	14.37	11.98	2.13
N	7	K-series	14.02	14.02	13.34	2.44
Ce	58	L-series	4.76	4.76	0.45	0.17
		Total:	100.00	100.00	100.00	

(B)

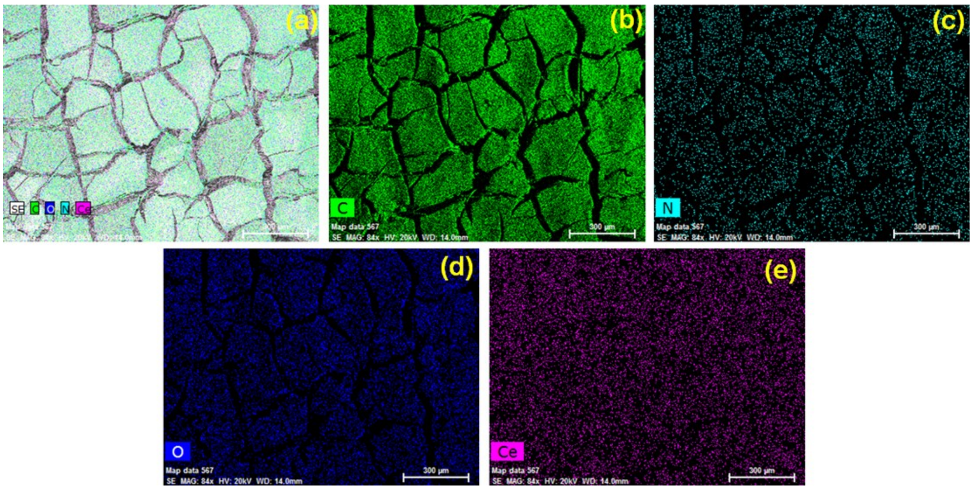

Figure SI10. (A) EDAX data of GNB–modified–CF electrode after the determination of Ce^{3+} by CV analysis in Acidic Buffer Solution (pH 4.0±0.05) at a scan rate of 50 mV/s; (B) Elemental mapping of GNB–modified–CF electrode after the determination of Ce^{3+} (a), C (b), N (c), O (d) and Ce (e).

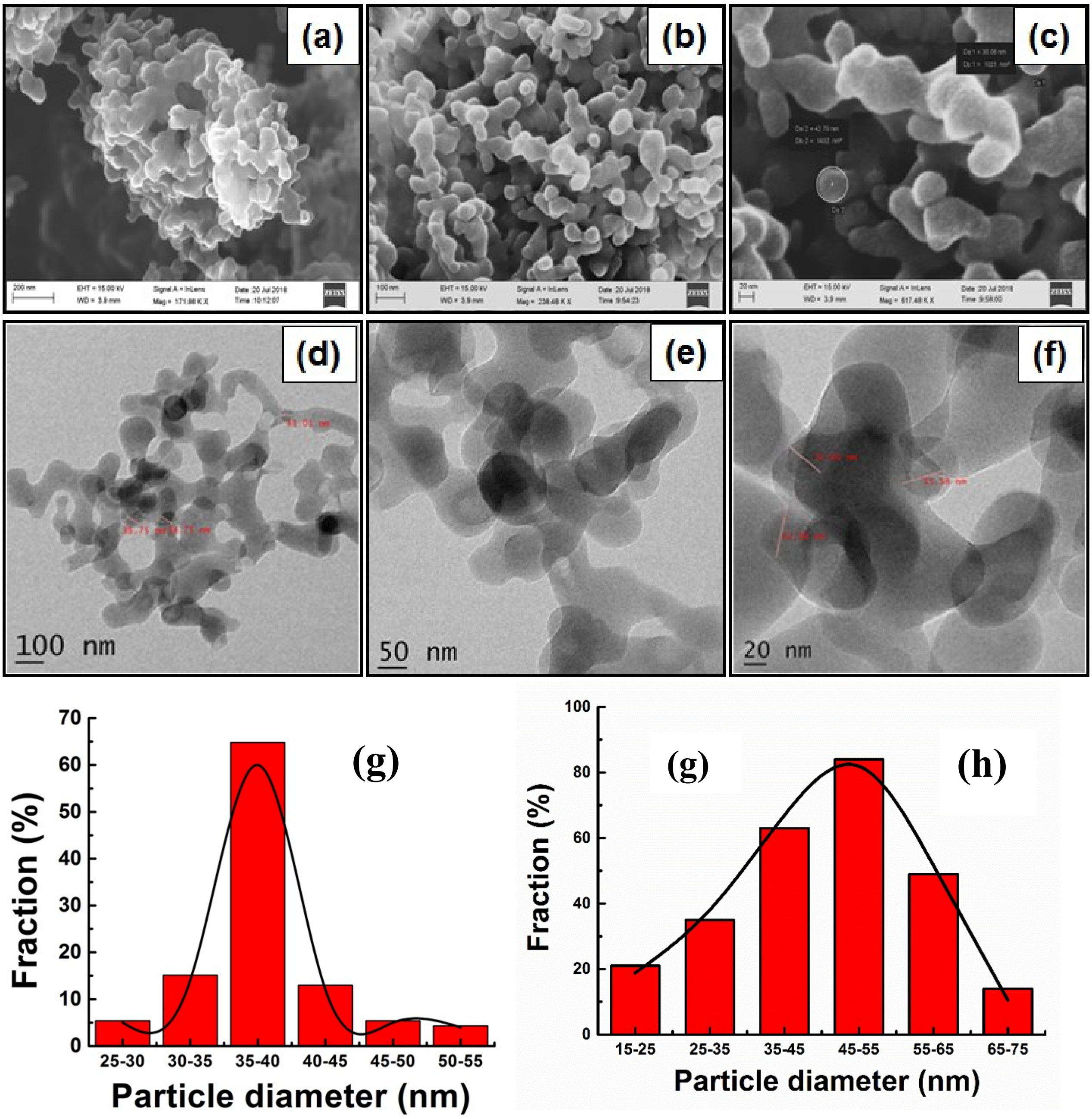

Figure SI11. FE-SEM (a-c) and HR-TEM (d-f) images of GNB clearly represents the formation of NBs with an average diameter of 35-40 nm. Scale bar (a) 200 nm, (b) 100 nm, (c) 20 nm, and (d) 100 nm, 50 nm, 20 nm. Size distributions of GNB before and after electrochemical

determination of Ce^{3+} (g, h, respectively). These image (a→g) are reprinted with permission from ACS Appl. Mater. Interfaces 11, 19339−19349 (2019).

5. XPS analysis

(A)

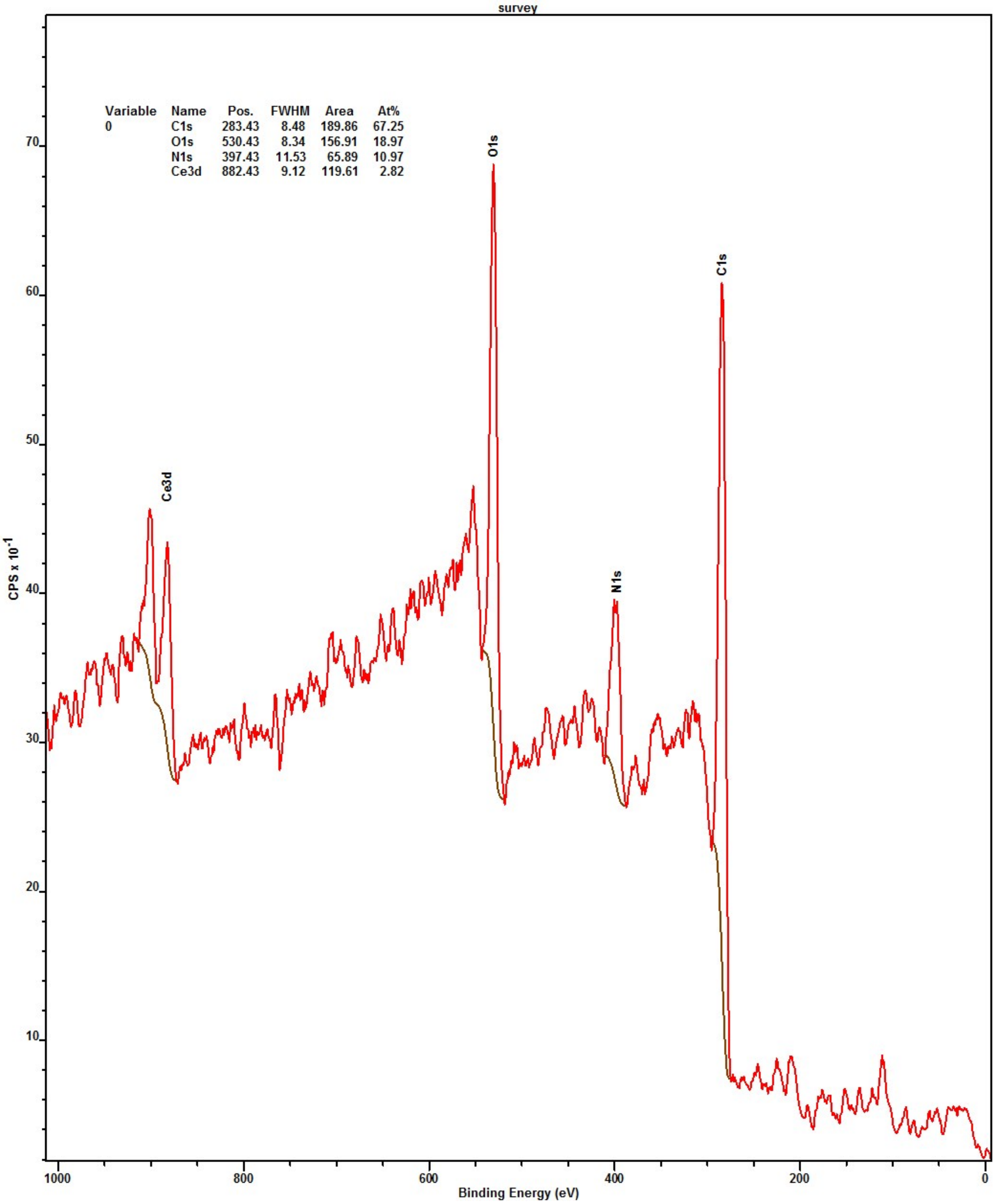

(B)

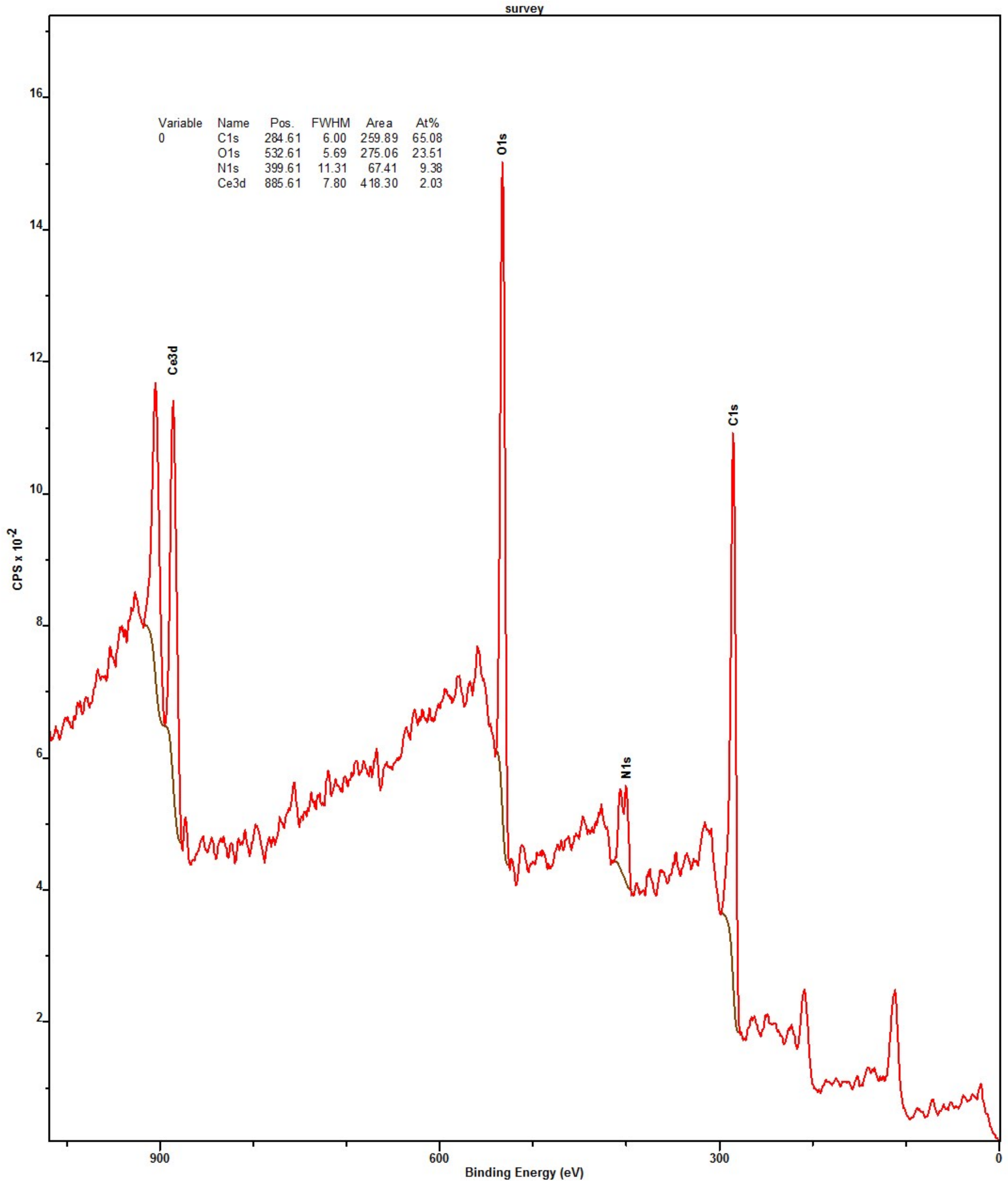

Figure SI12. X-ray Photoelectron survey spectrum of GNB–modified–CF electrode (A) and GNB–modified–ITO electrode (B) with at% details.

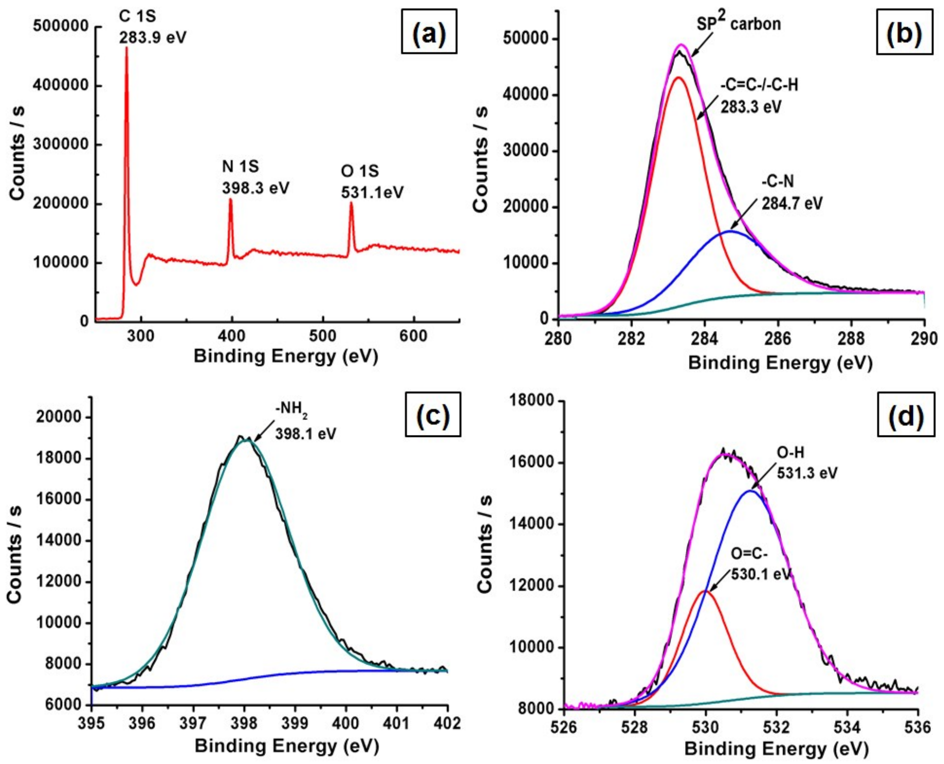

Figure SI13. X-ray Photoelectron Spectrum of GNB: survey spectrum (a); high resolution XPS of C 1s (b); N 1s (c); O 1s (d). These XPS image are reprinted with the permission from ACS Appl. Mater. Interfaces 11, 19339−19349 (2019).

6. Raman spectroscopy studies

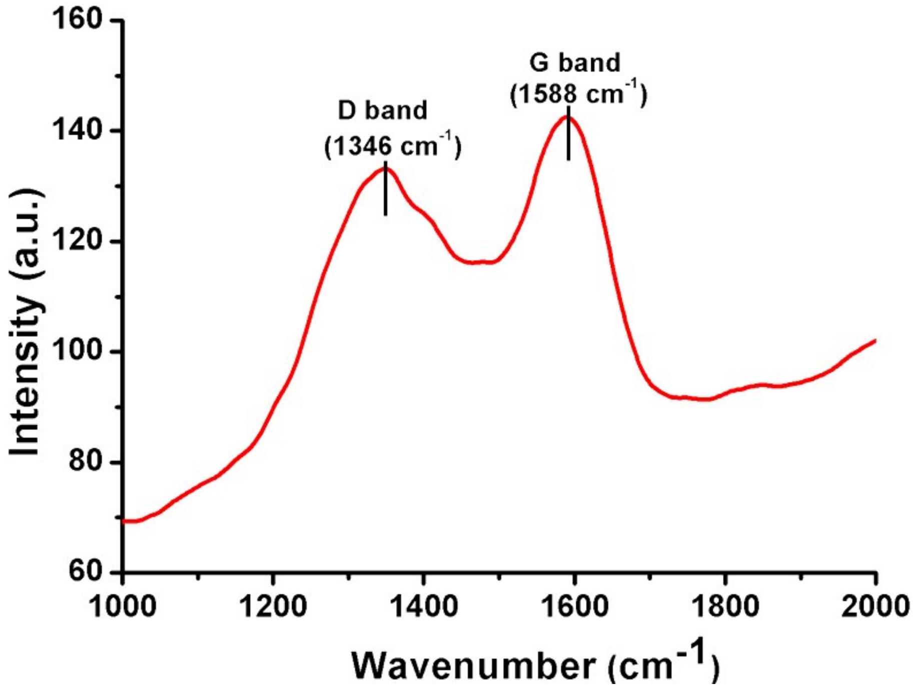

Figure SI14. Raman spectrum of as-prepared GNB.

Table SI2. Comparison of reported probes for Ce^{3+} detection so for.

**Publication details**	**Material**	**LoD**	**ToD**	**Method**
Sens. Actua. B. 174 (2012) 237-244	Schiff Base-Carbon Nanotube–Nanosilica–Ionic Liquid	6.45×10^{-9} M	5 sec	Potentiometry
Chem. Eng. J. 228 (2013) 327–335	4-Tert-Octyl-4-((Phenyl)Diazenyl)Phenol	0.18 lg/L	25 min	Adsorption
Chem. Eng. J. 265 (2015) 210–218	Organic Ligand Of 4-Dodecyl-6-((4-(Hexyloxy)Phenyl) Diazenyl) Benzene-1,3-Diol	0.12 lg/L	25 min	Adsorption
Spectrochimica Acta Part A 206 (2019) 240-245	Carbon Nanodots	0.7 µM	-	Fluorescence quenching
J. Fluoresc. 27 (2017) 331-338	Graphene Quantum Dots	3.8×10^{-7} mol L^{-1}	1 min	Fluorescence quenching
Biosens. Bioelectron. 68 (2015) 598-603	Gnps Synthesized Using The Extract Of Solanum Lycopersicums	2 to 50 ppm	10 min	UV<br>DLS
Talanta, 200 (2019) 249-255	Silver Sulfide Quantum Dots (Ag_2S) And Graphitic Carbon Nitride Nanosheets	6.4×10^{-8} mol L^{-1}	40 min	Fluorescence quenching
Mater. Lett. 227 (2018) 154–157	Graphene Oxide Resorcinol Hybrid Material	-	2 min	PET and ICT processes
Electro-analysis 21 (2009) 1605-1610	N'-[(2-Hydroxyphenyl) Methylidene]-2-Furohydrazide	0.8 nmol dm^{-3}	-	Voltammetric

J. Rare Earths, 28 (2010) 387-390	Tribromoarsenazo	5.1×10^{-8}m ol/L ng/mL	-	Spectrophotometric
*J. Electroanal. Chem***.** 808 (2018) 41-49	Electropolymerized Poly-Catechol and Ion-Imprinted Membrane	1×10^{-12} m ol L^{-1}	-	Adsorptive stripping voltammetry
Chemical Data Collections, 13–14 (2018) 28-39	6-{4-(2,4-Dihydroxy-Phenyl)Diazenyl)Phenyl}-2-Oxo-4-Phenyl-1,2-Dihydropyridine-3-Carbonitrile	0.6 ng $molL^{-1}$	-	Spectrophotometric
J. Fluores. 25 (2015) 1855-1866	Glycine Dithiocarbamate (GDTC)-Functionalized Manganese Doped Zns Quantum Dots (Qds)	2.3×10^{-7} $molL^{-1}$	-	Spectrophotometric
Electroanal. 29 (2017) 1124-1130	Indium Tin Oxide	5.8 nM	<1 min	Stripping voltammetric
Sens. Actua. B 191 (2014) 192–203	Thiol surfactant assembled on gold nanoparticles	3.25×10^{-10} mol L^{-1}	< 1 min	Potentiometry
**Current work**	**Functionalized Graphene Quantum Dots (DAN-GQDs)**	**2.60 µM**	**< 1 min**	**Electrochemical methods (CV and DPV)**

References

1. Yan, R.; Wu, H.; Zheng, Q.; Wang, J.; Huang, J.; Ding, K.; Guo, Q.; Wang, J. Graphene Quantum Dots Cut from Graphene Flakes: High Electrocatalytic Activity for Oxygen Reduction and Low Cytotoxicity. *RSC Adv.* **2014**, *4*, 23097–23106.
2. Maity, N.; Kuila, A.; Das, S.; Mandal, D.; Shit, A.; Nandi, A. K. Optoelectronic and Photovoltaic Properties of Graphene Quantum Dot-Polyaniline Nanostructures. *J. Mater. Chem. A* **2015**, *3*, 20736–20748.
3. Mei, B. A.; Munteshari, O.; Lau, J.; Dunn, B.; Pilon, L. Physical Interpretations of Nyquist Plots for EDLC Electrodes and Devices. *J. Phys. Chem. C* **2018**, *122*, 194–206.
